# Probing the Influence of Defects, Hydration, and Composition on Prussian Blue Analogues with Pressure

**DOI:** 10.1021/jacs.0c13181

**Published:** 2021-02-25

**Authors:** Hanna L. B. Boström, Ines E. Collings, Dominik Daisenberger, Christopher J. Ridley, Nicholas P. Funnell, Andrew B. Cairns

**Affiliations:** † Max Planck Institute for Solid State Research, Heisenbergstraße 1, D-70569 Stuttgart, Germany; ‡ Department of Inorganic Chemistry, Ångström Laboratory, Uppsala University, Box 538, SE-751 21 Uppsala, Sweden; § Department of Chemistry, University of Oxford, Inorganic Chemistry Laboratory, South Parks Road, Oxford OX1 3QR, U.K.; ∥ Centre for X-ray Analytics, EMPA - Swiss Federal Laboratories for Materials Science and Technology, Überlandstrasse 129, 8600 Dübendorf, Switzerland; ⊥ Diamond Light Source Ltd., Harwell Campus, Didcot OX11 0DE, U.K.; ¶ ISIS Neutron and Muon Source, Rutherford Appleton Laboratory, Harwell Campus, Didcot OX11 0QX, U.K.; # Department of Materials, Imperial College London, Royal School of Mines, Exhibition Road, London SW7 2AZ, U.K.; ∇ London Centre for Nanotechnology, Imperial College London, London SW7 2AZ, U.K.

## Abstract

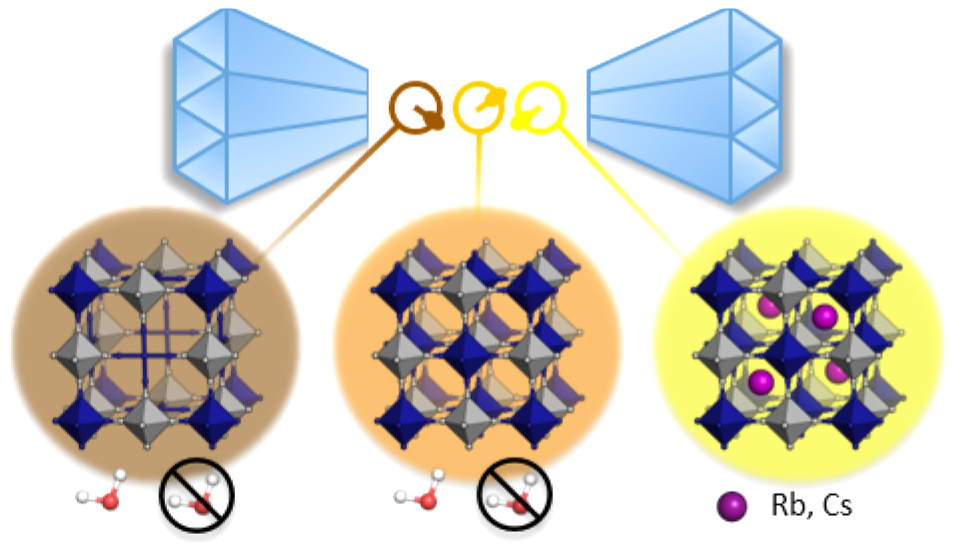

The vast compositional space of Prussian blue analogues (PBAs), formula A_*x*_M[M′(CN)_6_]_*y*_·*n*H_2_O, allows for a diverse range of functionality. Yet, the interplay between composition and physical properties—e.g., flexibility and propensity for phase transitions—is still largely unknown, despite its fundamental and industrial relevance. Here we use variable-pressure X-ray and neutron diffraction to explore how key structural features, i.e., defects, hydration, and composition, influence the compressibility and phase behavior of PBAs. Defects enhance the flexibility, manifesting as a remarkably low bulk modulus (*B*_0_ ≈ 6 GPa) for defective PBAs. Interstitial water increases *B*_0_ and enables a pressure-induced phase transition in defective systems. Conversely, hydration does not alter the compressibility of stoichiometric MnPt(CN)_6_, but changes the high-pressure phase transitions, suggesting an interplay between low-energy distortions. AMnCo(CN)_6_ (A^I^ = Rb, Cs) transition from *F*4̅3*m* to *P*4̅*n*2 upon compression due to octahedral tilting, and the critical pressure can be tuned by the A-site cation. At 1 GPa, the symmetry of Rb_0.87_Mn[Co(CN)_6_]_0.91_ is further lowered to the polar space group *Pn* by an improper ferroelectric mechanism. These fundamental insights aim to facilitate the rational design of PBAs for applications within a wide range of fields.

## Introduction

**Figure 1:**
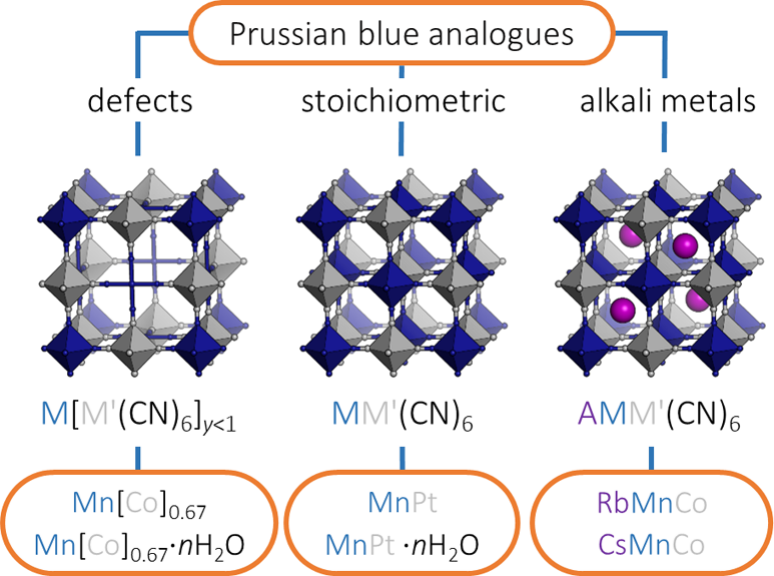
Different classes of Prussian blue analogues: defective M[M′(CN)_6_]_*y*<1_·*n*H_2_O, stoichiometric MM′(CN)_6_·*n*H_2_O and alkali-metal-containing A_*x*_M[M′(CN)_6_]_*y*_. The lower panel shows the systems investigated in this study.

Prussian blue analogues (PBAs), metal–cyanide frameworks with formula A_*x*_M[M′(CN)_6_]_*y*_·*n*H_2_O, show promise for a range of applications. This is partially attributed to the variable composition that allows for a diverse set of properties [Figure 1]. For example, introducing M′(CN)_6_ defects (*y* < 1) gives a percolating network of voids and so has potential for gas storage and catalysis.^1,2^ Alternatively, the possibility of inserting alkali metals on the A-site (*x* > 0) enables the application of PBAs as positive electrodes in secondary Na-ion batteries.^3,4^ Thus, the functionality can be optimized via crystal engineering. In addition, it is crucial to consider how such compositional changes influence the physical properties of PBAs, e.g., mechanical stability, phase behavior, and flexibility. These features are equally important considerations for the development of functional PBAs. The simple average crystal structure belies this compositional and structural complexity as most PBAs—whether defective or alkali-metal-containing or neither—crystallize in face-centered cubic space groups.^5−7^ Thus, a different probe is needed to fully understand these materials.

Studying materials under nonambient conditions is a useful tool to explore key structural interactions at play in materials. For example, variable-temperature studies of PBAs have given insight into the relationship between composition and flexibility. The flexibility of the MPt(CN)_6_ series depends on the radius of M, as larger cations bind more weakly to the nitrogen atom, leading to an enhanced propensity for transverse vibrations.^8^ This underpins the trend in negative thermal expansion (NTE) for this series of PBAs and CdPt(CN)_6_ correspondingly shows the greatest NTE.^8^ Likewise, the impact of A-site cations on the lattice dynamics was demonstrated for Na_*x*_GaFe(CN)_6_, where increasing *x* inhibits the transverse phonons and thereby reduces the magnitude of the NTE.^9^ Such studies aid the understanding of the role of the metal identity and stoichiometry; however, they are limited by the relatively small structural variations induced by temperatures routinely available. For example, the soft phonons responsible for the thermal expansion do not condense upon cooling.^10−12^ As a result, displacive phase transitions that may give further insight are extremely rare.^13^ This is a stark contrast to the wealth of phase transitions—driven by octahedral tilting—in the related family of perovskites.^14,15^

Unlike temperature, hydrostatic pressure can be varied over many orders of magnitude and may provide vital information about the degrees of freedom of a system.^16^ As an illustration, the metal–organic framework (MOF) ZIF-8 readily absorbs molecules with effective diameter larger than the pore window.^17,18^ The apparent inconsistency was rationalized by a rotation of the imidazolate linkers—a distortion which had been observed by high-pressure X-ray diffraction using a penetrating pressure-transmitting medium (PTM).^19^ This demonstrates how crystallography under extreme conditions can aid the understanding of functionally relevant phenomena at near-ambient conditions.

The crystallography of PBAs under high pressure is still relatively unexplored,^20−23^ yet it is expected to reveal similar insights into the fundamental structural behavior. For example, pressure-induced phase transitions can provide information about the accessible low-energy distortion pathways. Mechanical stability can also be probed, which is essential for many applications.^24^ In some cases, pressure can change the electronic structure and thereby give rise to phenomena such as magnetic pole inversions,^25^ increase of the magnetic ordering temperature,^26^ or spin crossover transitions.^27−29^ Likewise, the insertion of Na cations during electrochemical cycling exerts chemical pressure on the framework and this is accompanied by phase transitions.^30,31^ Thus, a deeper understanding of the structural pressure response may allow for optimization of these properties.

Moreover, PBAs constitute suitable candidate materials for the investigation of structure–pressure behavior relationships in the wider families of coordination polymers (e.g., metal–organic frameworks, MOFs) and other porous frameworks (e.g., zeolites). The tunable pore volume and compositional versatility allows for systematic exploration of factors such as defects and pore contents (alkali metals or water). The high symmetry of PBAs facilitates solving high-pressure phases using powder diffraction. In addition, the M:M′ ratio provides a direct measure of the number of defects, which is in contrast to MOFs, where the quantification and characterization of defects is a considerable challenge.^32^ Nevertheless, defects are a crucial tool for property optimization in MOFs,^32−34^ and there is an emerging interest in their effect on the pressure response.^35−37^ The pressure behavior of PBAs may therefore have ramifications for other types of materials.

This manuscript uses variable-pressure X-ray and neutron diffraction (XRD/ND) to explore how the key structural features of defects, hydration, and composition affect the pressure behavior of PBAs. The interplay between structural features in this ostensibly simple family means that untangling the role of each can be challenging, yet by selecting appropriate systems, we obtain a fundamental insight that will benefit the development of functional PBAs. The systems under study along with their abbreviations are summarized in Table 1 and comprise PBAs with diverse stoichiometries. Our study starts by comparing the results for Mn[Co(CN)_6_]_0.67_ and MnPt(CN)_6_, revealing that defects exert a strong effect on the elastic properties and phase transitions. Subsequently, the role of hydration is discussed and how water influences the pressure-induced phase behavior for both systems, despite the ambient structures being unaffected by the hydration state. Lastly, a comparison of RbMnCo(CN)_6_ and CsMnCo(CN)_6_ indicates that the radius of the A-site cation affects the onset of the pressure-induced phase transition from *F*4̅3*m* to *P*4̅*n*2. RbMnCo(CN)_6_ undergoes further symmetry lowering to the polar space group *Pn* at 1 GPa, driven by an improper ferroelectric mechanism. This presents a very rare example of a polar PBA. We conclude by discussing the results in the context of other coordination polymers and MOFs.

**Table 1:** An Overview of the Prussian Blue Analogues in This Study, Including the Composition, Space Group, Phase Transition Pressure (*p*_T_), the Bulk Modulus (*B*_0_) from a Second-Order Birch–Murnaghan Fit,^38,39^ and the Radiation Used^a^

abbreviation	composition	space group	*p*_T_ (GPa)	*B*_0_ (GPa)	radiation
**Defective**					
	Mn[Co(CN)_6_]_0.67_·*n*H_2_O*^23^	*Fm*3̅*m*	1.46(13)	13.5(6)	X-ray
Mn[Co]_0.67_·*n*D_2_O	K_0.06(2)_Mn[Co(CN)_6_]_0.76(3)_·*n*D_2_O	*Fm*3̅*m*	–	15.18(6)	neutron
Mn[Co]_0.67_	K_0.06(2)_Mn[Co(CN)_6_]_0.76(3)_	*Fm*3̅*m*	–	6.5(7)	X-ray
Cd[Co]_0.67_	K_0.1(2)_Cd[Co(CN)_6_]_0.75(6)_	*Fm*3̅*m*	–	6.5(4)	X-ray
**Stoichiometric**					
MnPt·*n*H_2_O	MnPt(CN)_6_·*n*H_2_O^23^	*Fm*3̅*m*	1.31(10)	35(2)	X-ray
MnPt·*n*D_2_O	MnPt(CN)_6_·*n*D_2_O	*Fm*3̅*m*	–	31.9(9)	neutron
MnPt	MnPt(CN)_6_	*Fm*3̅*m*	0.97(12)	33(2)	X-ray
FePt	FePt(CN)_6_^29^	*Fm*3̅*m*	–	33(5)	X-ray
**Alkali-containing**					
RbMnCo	Rb_0.87(4)_Mn[Co(CN)_6_]_0.91(3)_	*F*4̅3*m*	0.23(5)	20	neutron
RbMnCo-II	Rb_0.87(4)_Mn[Co(CN)_6_]_0.91(3)_	*P*4̅*n*2	0.94(6)	10.7(4)	neutron
RbMnCo-III	Rb_0.87(4)_Mn[Co(CN)_6_]_0.91(3)_	*Pn*	–	11.9(3)	neutron
CsMnCo	Cs_1_Mn[Co(CN)_6_]_1.00(4)_	*F*4̅3*m*	1.94(15)	31(2)	X-ray
CsMnCo-II	Cs_1_Mn[Co(CN)_6_]_1.00(4)_	*P*4̅*n*2	–	14.7(8)	X-ray

a The transition pressure is calculated as the average between the pressure before and after the transition. Asterisks denote single crystals and Roman numerals different phases. Isostructural systems from refs (23), (29) are also included. Note that the bulk modulus of RbMnCo is calculated based on two data points.

## Methods

Prussian blue analogues were synthesized as polycrystalline powders by dropwise addition of an aqueous solution of the appropriate metal salt—MnSO_4_, Mn(NO_3_)_2_, or Cd(OAc)_2_—to an aqueous stoichiometric solution of K_3_Co(CN)_6_ or K_2_Pt(CN)_6_. In the case of Cs- or Rb-containing systems, CsCl/RbCl was added to the K_3_Co(CN)_6_ solution in at least 10-fold molar excess. The reaction mixture was stirred for 2 h and the products isolated as micron-sized fine powders^40^ by filtration or centrifugation.

The compositions of CsMnCo and Mn[Co]_0.67_ were analyzed by inductively coupled plasma (ICP) using a PerkinElmer ICP-OES Avio 200 spectrometer. The samples were heated to 600 °C for 6 h prior to dissolution in aqua regia and dilution using ICP grade water. The plasma was ignited using Ar gas and the instrument allowed to thermally stabilize for 5 min. The results were averaged over three measurements. Cs cannot be detected by ICP, but its concentration was inferred by charge balance. Elemental analysis for RbMnCo was carried out by Medac Ltd., and the uncertainty calculated as the standard deviation between the two batches used for the ND experiment. The composition of Cd[Co]_0.67_ was confirmed by energy-dispersive X-ray spectroscopy in a Zeiss Merlin scanning electron microscope equipped with an Ultim Max 100 mm^2^ Silicon Drift Detector. The data were acquired using an acceleration voltage of 20 kV and for 50 s at a working distance of 6.7 mm. Sixteen sample positions were probed, and the results averaged with uncertainty calculated as the standard deviation. The data were evaluated using the AZtec software, and light elements (C, N, and O) were included in the fit to the spectra.

In-house X-ray diffraction of RbMnCo was carried out on a Bruker D8 Advance diffractometer using Cu radiation and a PSD-strip detector Lynxeye XE. Variable-pressure powder X-ray diffraction of dehydrated samples Mn[Co]_0.67_, Cd[Co]_0.67_, MnPt, and CsMnCo) was performed on the I15 beamline at Diamond Light Source, U.K. at an energy of 30 keV. All samples were dried at 80 °C under a vacuum for >24 h prior to being loaded into a diamond anvil cell (DAC) inside a glovebox, with Daphne 7373 oil as a pressure-transmitting medium and a ruby for pressure calibration.

Variable-pressure neutron powder diffraction was carried out on the PEARL diffractometer^41^ at the ISIS Neutron and Muon source, U.K. Mn[Co]_0.67_·*n*D_2_O and MnPt·*n*D_2_O were stirred in D_2_O overnight, excess D_2_O was removed by centrifugation, and the mixture was dried overnight at 60 °C. For each sample, the ground powder was loaded in a null-scattering TiZr gasket, along with a Pb pressure marker, and placed between single-toroid ZrO_2_-toughened Al_2_O_3_ anvils. The gasket and anvil assembly was precooled to ca. 5 °C in order to load perdeuterated pentanes as a PTM; the cooling minimized any loss through evaporation. The anvil assembly was then loaded in a V3 Paris-Edinburgh press and mounted in the PEARL instrument. Pressure was controlled via an oil-driven piston. RbMnCo was dehydrated under a vacuum at 120 °C for 24 h and was loaded as described above, except that the pressure-transmitting medium was a perdeuterated methanol–ethanol mixture, in a 4:1 volume ratio.

Analysis of powder diffraction patterns was carried out by Pawley and Rietveld refinements using the software TOPAS.^42−44^*hkl*-dependent peak broadening was applied to account for the anisotropic peak shapes in CsMnCo caused by Cs disorder. The alkali cation occupancies in CsMnCo and RbMnCo were refined on the two crystallographically inequivalent sites in *F*4̅3*m*, subject to the constraint that total occupancy agreed with the elemental analysis. For RbMnCo, the cation distribution was refined in a XRD pattern collected at ambient conditions and subsequently fixed in refinements of the neutron diffraction patterns. The high-pressure phases of RbMnCo and CsMnCo were refined using symmetry modes with the software ISODISTORT.^45^ To avoid overparametrization, only modes corresponding to the primary order parameters (the tilts Γ_4_^+^ and X_3_^+^) and the symmetric strain (Γ_1_^+^) were refined in the monoclinic phase of RbMnCo. All fits to data, refined lattice parameters and further details are presented in the Supporting Information; crystallographic data for previously unreported structures have been deposited to the CCDC/FIZ-Karlsruhe with reference numbers CSD 2048874–2048878.

The variable-pressure unit cell lattice parameters were fitted using the second-order Birch–Murnaghan equations of state as implemented in the software EoSfit-GUI.^38,39,46^ The fits to the *p*–*V* data and normalized pressure vs Eulerian strain plots are provided as Supporting Information. In the case of CsMnCo, a third-order fit was also performed, the results of which are discussed in the SI.

## Results

To explore a range of stoichiometries, hydrated and dehydrated PBAs with nominal compositions M[Co(CN)_6_]_0.67_ (M = Mn, Cd), MnPt(CN)_6_, and AMnCo(CN)_6_ (A = Rb, Cs) were studied. The experimentally determined compositions and abbreviations used are shown in Table 1; note that (i) the inclusion of A-site cations and defects act in competition during the synthesis, which typically leads to some coexistence,^47,48^ and (ii) while the water content of our dehydrated samples is clearly lower than for the as-synthesized samples, determination of the exact hydration state is extremely challenging and therefore outside the scope of this study. The effects of defects, hydration, and composition on the high-pressure results will now be discussed in turn.

### The Role of Defects

**Figure 2:**
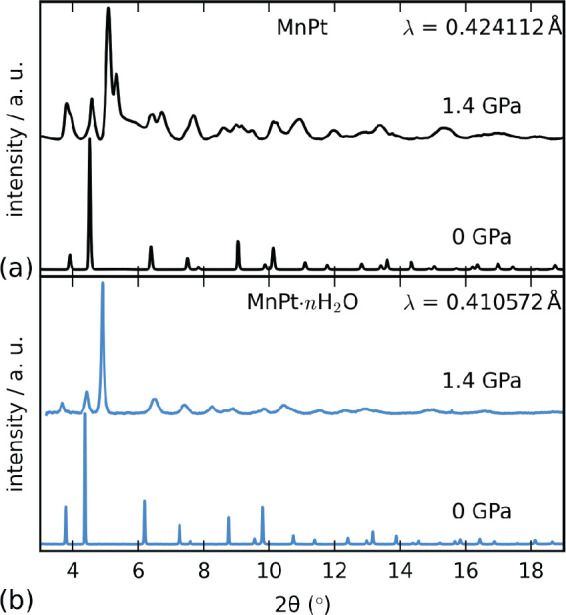
Background-subtracted XRD patterns of (a) MnPt and (b) MnPt·*n*H_2_O at 0 and 1.4 GPa. Data for MnPt·*n*H_2_O were reported in ref (23).

M′(CN)_6_ defects reduce the connectivity and, critically, reduce resistance to mechanical compression and should therefore strongly affect the pressure behavior. To investigate this, we studied dehydrated Mn[Co]_0.67_ and MnPt using XRD up to ∼2 GPa. All patterns of Mn[Co]_0.67_ can be fitted with the ambient *Fm*3̅*m* structure, although the peaks broaden substantially upon compression, which suggests the onset of pressure-induced amorphization [Figure S1–S2]. Similar behavior was observed for Cd[Co]_0.67_ [Figure S3]; however, despite applying up to 4 GPa to Cd[Co]_0.67_, complete amorphization is not observed. For MnPt, additional reflections emerge at 1.08 GPa, suggesting a phase transition [Figure S4]. The high-pressure phase persists to 2.4 GPa and decompression returns the ambient cubic structure. Interestingly, the high-pressure phase differs from the rhombohedral high-pressure structure of *hydrated* MnPt·*n*H_2_O reported previously [Figure 2],^23^ but the large peak width prevents a structural solution.

The bulk moduli of Mn[Co]_0.67_ and MnPt were calculated by second-order Birch–Murnaghan^38,39^ fits as 6.5(7) GPa and 33(2) GPa, respectively [Table 1]. Cd[Co]_0.67_ has a bulk modulus of 6.5(4) GPa, implying that the metal substitution does not noticeably affect the mechanical properties for defective PBAs. The very low bulk modulus of the defective systems is in the same category as some highly porous MOFs and small-molecule organics.^49−53^ Likewise, MnPt and the isostructural FePt(CN)_6_ feature identical compressibilities,^29^ again suggesting that the rigidity is not influenced by the metal composition. Thus, stoichiometry rather than metal identities appears to dominate the bulk elastic response of Prussian blue analogues.

**Figure 3:**
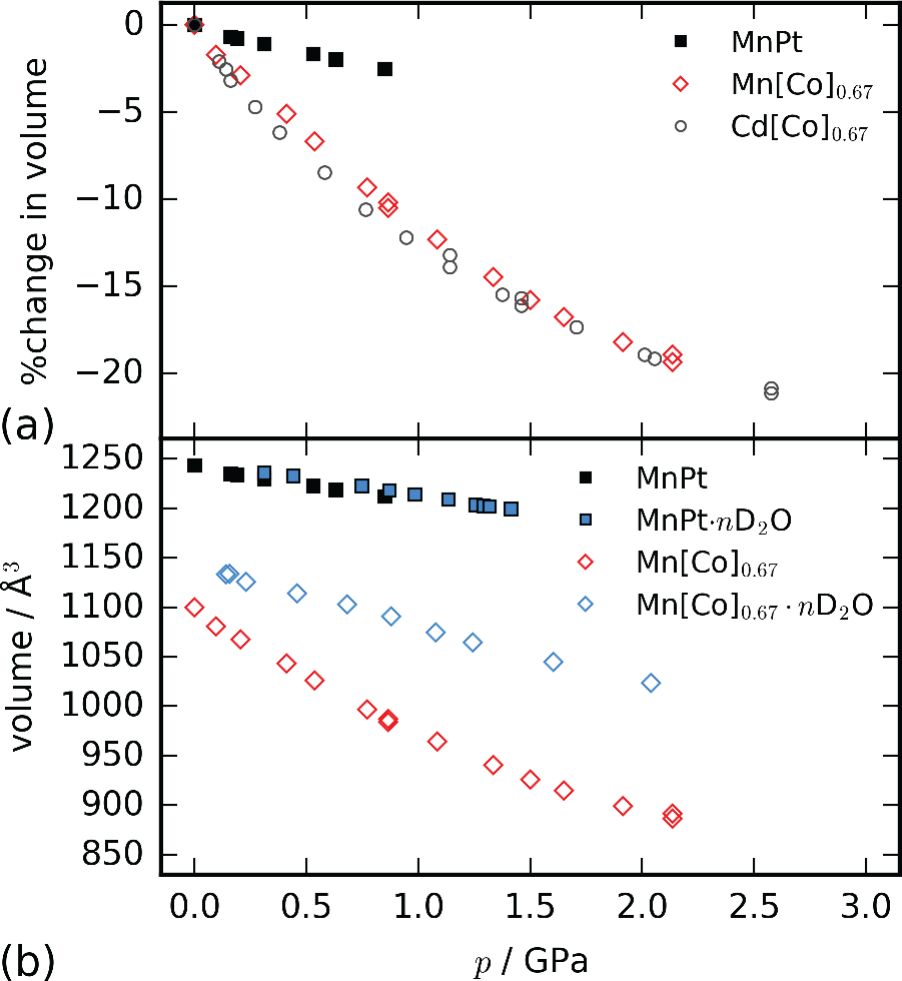
(a) The percentage change in the unit cell volume of defective and stoichiometric PBAs. (b) The cell volume as a function of pressure for MnPt and Mn[Co]_0.67_. Empty symbols are used for defective systems and hydrated compounds are denoted with blue markers. Errors in *y* are smaller than the data markers and pressure errors are within 0.1 GPa.

Further, the contrasting pressure responses of Mn[Co]_0.67_ and MnPt highlight how defects additionally prevent pressure-induced phase transitions [Figure 3(a)]. The defects increase the free pore space and disrupt the connectivity of the metal–cyanide framework. This allows for direct compression of octahedral columns into the voids,^54^ which enhances the compressibility. This local framework distortion is consistent with the peak broadening and loss of intensity of the defective PBAs upon compression. Hence, defective systems are likely to amorphize at lower pressures than the stoichiometric analogues. The additional degrees of freedom due to the voids mean that volume reduction can proceed by local rather than global distortions. While in this study the low solidification pressure of the PTM used (Daphne Oil) prevented further investigation of amorphization at higher pressure, this remains an exciting area for further work. Conversely, the full connectivity of MnPt increases the bulk modulus, and beyond a certain pressure threshold, a phase transition is necessary to decrease the volume. Thus, while the ambient average structure is insensitive to the presence of defects, their effect is clearly observed in the variable-pressure behavior.

### The Role of Hydration

To investigate the role of hydration, variable-pressure neutron diffraction patterns were collected up to ∼2.5 GPa for Mn[Co]_0.67_·*n*D_2_O and MnPt·*n*D_2_O using a Paris–Edinburgh (P–E) press [Figure S5–S6]. To the best of our knowledge—while a useful complementary tool to XRD due to the ability to, e.g., confirm or refine the presence of D_2_O within the structure, refine the binding orientation of cyanide—only one previous high-pressure neutron diffraction study of PBAs has been reported to date.^55^ From our diffraction data, we were able to confirm the presence of D_2_O in both samples using the structural model of ref (56). The bulk moduli were calculated as 15.18(6) and 31.9(9) GPa for Mn[Co]_0.67_·*n*D_2_O and MnPt·*n*D_2_O,  respectively [Table 1], and are consistent with the results from a high-pressure XRD study.^23^ For Mn[Co]_0.67_·*n*D_2_O, this represents a doubling of the bulk modulus compared to the dehydrated system discussed above, whereas no significant change is observed for MnPt [Figure 3(b), Table 1].

The discrepant effects of hydration on the compressibilities of Mn[Co]_0.67_·*n*D_2_O and MnPt·*n*D_2_O can be rationalized by considering the role of water in each compound. Water molecules in defective Mn[Co]_0.67_·*n*D_2_O bind to the coordinatively unsaturated Mn^II^ ions,^56^ and thus inhibit the compression of octahedral columns on opposing sides of a defect. Indeed, investigations of the local structure of Mn[Co(CN)_6_]_0.67_·*n*H_2_O show that coordinated water molecules decrease the flexibility of the framework.^57^ Conversely, water molecules in MnPt are interstitial and—as hydration has no effect on the bulk modulus—do not appear to impact the transverse vibrations responsible for framework strain to any great extent.^58^ It follows that the influence of hydration on the compressibility is dependent on the presence of defects.

The phase behavior of Mn[Co]_0.67_·*n*D_2_O and MnPt·*n*D_2_O in our neutron diffraction experiments contrasts with results from a previous XRD study.^23^ The neutron diffraction patterns of Mn[Co]_0.67_·*n*D_2_O, collected using a P–E press, show reflection broadening at 0.44 GPa and the Bragg reflections vanish at 2 GPa. Likewise, MnPt·*n*D_2_O becomes amorphous at 1.4 GPa, albeit with less broadening than Mn[Co]_0.67_·*n*D_2_O. Crystallinity is recovered upon decompression, indicating that local order is retained and that compression, rather than radiation, is the cause of the amorphization. Amorphization also occurred during compression in fluorinert instead of pentanes, which suggests that the amorphization is not due to the choice of PTM. Although pressure-induced amorphization (PIA) is well-known for other framework systems,^50,59,60^ a previous XRD study using a diamond anvil cell (DAC) showed retention of crystallinity for a single crystal of Mn[Co(CN)_6_]_0.67_·*n*H_2_O and a powder specimen of MnPt·*n*H_2_O up to 2 GPa.^23^ In the absence of a more definitive explanation, we suggest that this discrepancy may result from the larger sample volume used in the P–E press, which will enhance the effect of grain–grain interactions (see Supporting Information for further discussion).^61^

In contrast, these PBAs underwent pressure-induced *Fm*3̅*m*–*R*3̅ transitions in the XRD study,^23^ resulting from octahedral tilting (tilt system *a*^–^*a*^–^*a*^–^ in Glazer notation).^14,62^ The behavior reported in ref (23) is considered more representative of the true pressure-induced behavior of the hydrated PBAs and will be used in subsequent discussions. Interestingly, the phase changes of the hydrated materials contrasts with the behavior of the dehydrated frameworks discussed above. For Mn[Co]_0.67_, water is a necessary condition for the phase transition to occur, as the dehydrated Mn[Co]_0.67_ remains cubic up to 2 GPa. It is possible that the water forms a hydrogen-bonded network that bridges the defects and facilitates correlated framework distortions. In addition, the coordination sphere of Mn^II^ may (locally) change from octahedral to tetrahedral upon dehydration, as has been observed for Co[Co(CN)_6_]_0.67_.^63^ MnPt exhibits phase transitions upon compression regardless of its hydration state;^23^ however, the high-pressure structures are different [Figure 2].

### The Role of Composition

RbMnCo and CsMnCo were chosen to study the effect of composition, as the identity of extra-framework (A-site) cations presumably exerts the largest effect on the structural behavior. Both samples were dehydrated prior to the diffraction experiment, but as they are not considered porous, the effect of hydration is less relevant than for MnPt and Mn[Co]_0.67_. RbMnCo and CsMnCo were investigated with neutron and X-ray radiation, respectively [Figure S7–S10]. RbMnCo retained its crystallinity to higher pressures than MnPt·*n*D_2_O and Mn[Co]_0.67_·*n*D_2_O discussed above, which may result from its higher density or the lack of interstitial water. At ambient conditions, both systems crystallize in the noncentrosymmetric space group *F*4̅3*m*, as a result of occupational A-site cation order.^6,64^ Although the ND patterns for RbMnCo cannot discriminate between *F*4̅3*m* and *Fm*3̅*m* due to the relatively lower neutron scattering length of Rb, XRD data indicate a preference for *F*4̅3*m* [Figure S8].

The bulk modulus of the ambient phase of CsMnCo in the pressure range 0–1.6 GPa was calculated as *B*_0_ = 31(2) GPa from a second-order Birch–Murnaghan fit. This is similar to *B*_0_ of MnPt, suggesting that the incorporation of extra-framework cations does not significantly affect the compliance of the cubic phase—in contrast to some other framework materials.^65−67^ In the region 1.6–2.0 GPa, CsMnCo appears to undergo pressure-induced softening and gradually transitions into a tetragonal phase around 2 GPa. Due to the gradual nature of the transition, it is difficult to pinpoint the exact transition pressure given the resolution of the data. A third-order Birch–Murnaghan fit in the range 0–2 GPa is presented and discussed in the SI. A linear fit to data for RbMnCo estimated *B*_0_ ≈ 20 GPa using the software PASCal,^68^ yet this value should be treated with caution due to the few data points available.

**Figure 4:**
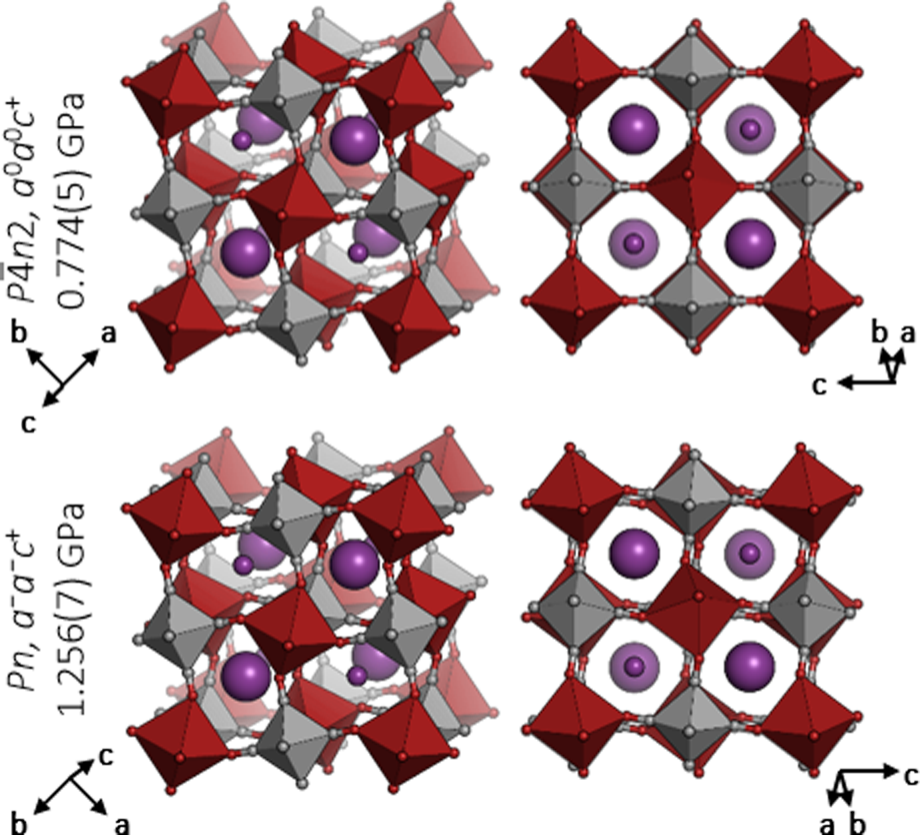
Crystal structures of the tetragonal and monoclinic high-pressure phases of RbMnCo. The structures are rotated to ensure a consistent viewing direction.

RbMnCo and CsMnCo remain cubic until 0.23(5) GPa and ca. 2 GPa, respectively, where phase transitions to the tetragonal space group *P*4̅*n*2 (phase II) occur. The symmetry lowering is driven by in-phase octahedral tilting along one axis (*a*^0^*a*^0^*c*^+^),^14^ as illustrated in Figure 4. In both cases this phase transition occurs below the hydrostatic limit of the PTM used.^69,96^ This tilt system is precedented by the high-pressure phase of RbMnFe(CN)_6_,^22^ but is rare in double oxide perovskites.^70^ Given that the X-ray scattering of CsMnCo is dominated by the metals, the sensitivity to the cyanide positions is poor. Therefore, an alternative structure with tilt system *a*^0^*a*^0^*c*^–^ and space group *I*4̅ gives a very similar fit to the data [Figure S21]. However, based on a very weak reflection not well accounted for in the *I*-centered space group and the fact that *I*4̅ symmetry is yet unreported in PBAs—albeit common in oxide perovskites^70^—the *P*4̅*n*2 structure is considered more likely. Nonetheless, it is clear that the presence of a large alkali metal changes the high-pressure phases compared to Mn[Co(CN)_6_]_0.67_·*n*H_2_O and MnPt·*n*H_2_O.^23^

The critical pressures of the *Fm*3̅*m*–*P*4̅*n*2 transition of RbMnCo and CsMnCo differ by nearly an order of magnitude. Thus, the framework instability of RbMnCo is more accessible than the corresponding distortion of CsMnCo or indeed of MnPt or Mn[Co]_0.67_. This *a*^0^*a*^0^*c*^+^ tilt in RbMnCo-II also features at ambient conditions in the Jahn–Teller-distorted RbCuM′(CN)_6_ (M′ = Fe^III^, Co^III^).^40,71^ The low-pressure soft mode distortion in RbMnCo can be attributed to the higher charge density of Rb^I^ compared to Cs^I^. This provides a greater electrostatic attraction to the cyanide linkers and should therefore lower the transition pressure. Accordingly, KMnCo(CN)_6_ or NaMnCo(CN)_6_ may feature even lower critical pressures. As the onset of tilting both improves the conductivity by narrowing the band gap,^72^ and enhances the magnetic ordering temperature,^26^ identifying driving forces for tilting is crucial for property optimization.

At 0.94(6) GPa, RbMnCo-II undergoes further symmetry lowering to the monoclinic space group *Pn* (phase III). This transition was not observed for the Cs analogue, but it is possible that it occurs beyond the investigated pressure range. The *Pn* phase of RbMnCo persists up to ∼4 GPa, but reflections broaden substantially upon compression in the P–E press, as for MnPt· *n*D_2_O and Mn[Co]_0.67_·*n*D_2_O. As a result, symmetry-mode Rietveld refinements were only carried out up to 2.5 GPa and only modes corresponding to strain and primary order parameters were refined. Monoclinic RbMnCo-III exhibits *a*^–^*a*^–^*c*^+^ tilts,^14^ which also occur at ambient conditions in several PBAs with formula A_2_MM′(CN)_6_.^73−75^ These systems crystallize in centrosymmetric *P*2_1_/*n*, whereas the Rb occupational order present in RbMnCo yields the polar subgroup *Pn*. As PBAs normally adopt high-symmetry cubic space groups, polar PBAs are extremely rare. The monoclinic phase of RbMnCo is not reported for the analogous RbMnFe(CN)_6_ under pressure,^22^ and further investigations of RbMnM′(CN)_6_ (M′ = Fe^III^, Co^III^) would be of interest. The results for RbMnCo and CsMnCo demonstrate the range of interesting structures of alkali-metal-containing PBAs accessible by modest pressures.

**Figure 5:**
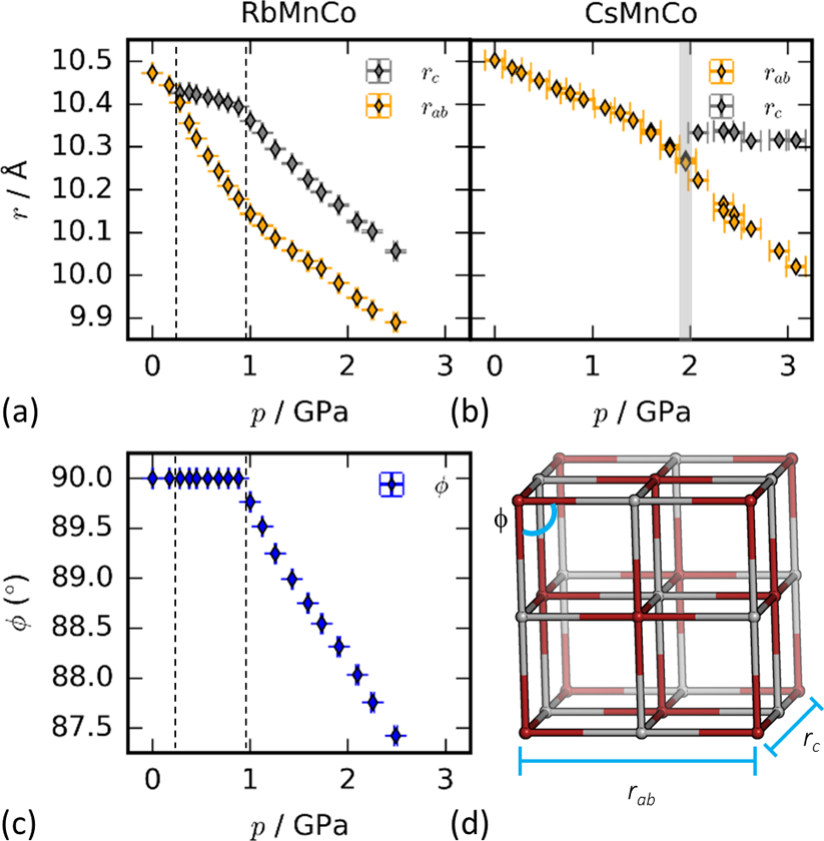
Pressure dependence of the strut lengths *r*_*ab*_ and *r*_*c*_ for (a) RbMnCo and (b) CsMnCo and of (c) the hinging angle ϕ for RbMnCo. The transition pressures are denoted by vertical dashed lines or a gray rectangle. (d) The parametrization of the PBA structure in terms of the mechanical building units.

The tilted high-pressure phases RbMnCo-II, RbMnCo-III, and CsMnCo-II are more compressible than cubic PBAs. Their bulk moduli were calculated as 10.7(4), 11.9(3), and 14.7(8) GPa, respectively, which for CsMnCo represents a reduction by ∼1/2 relative to the ambient phase. To compare their axial compressibilities, it is helpful to use the mechanical building unit approach,^76^ whereby the PBA structure is parametrized by strut lengths *r*_*ab*_ and *r*_*c*_ and angle ϕ [Figure 5, Table S8]. Principal axes compressibilities are given in Table S9. For cubic structures, *r*_*ab*_ = *r*_*c*_ = *a* and ϕ = 90°. In tetragonal and monoclinic symmetry, the strut lengths *r*_*c*_ and *r*_*ab*_ correspond to the Mn–Mn distance along *c* and in the *ab* plane, respectively. The angle ϕ represents the Mn–Mn–Mn angle in the *ab* plane and only deviates from 90° in the monoclinic phase. The tetragonal phases show a relatively compliant *r*_*ab*_ and a considerably stiffer *r*_*c*_ [Table 2]. This is consistent with the main mechanism for compression being increasing tilting, which only contracts the *ab* plane. The greater softness along *c* of RbMnCo-II compared to the Cs analogue—5.3(6) TPa^–1^ vs 2.4(6) TPa^–1^—may relate to the soft tilt mode that later condenses to drive the tetragonal–monoclinic transition. In monoclinic RbMnCo-III, the presence of tilts in all three directions results in similar compressibilities of *r*_*ab*_ and *r*_*c*_, and the framework starts to hinge in the *ab* plane.

**Table 2:** Linear Compressibilities of the High-Pressure Phases of RbMnCo and CsMnCo

	compressibilities (TPa^–1^)		
phase	*r*_*ab*_	*r*_*c*_	ϕ
CsMnCo-II	19.0(9)	2.4(6)	
RbMnCo-II	36(2)	5.3(6)	
RbMnCo-III	16.9(4)	19.9(5)	17.4(3)

## Discussion

**Figure 6:**
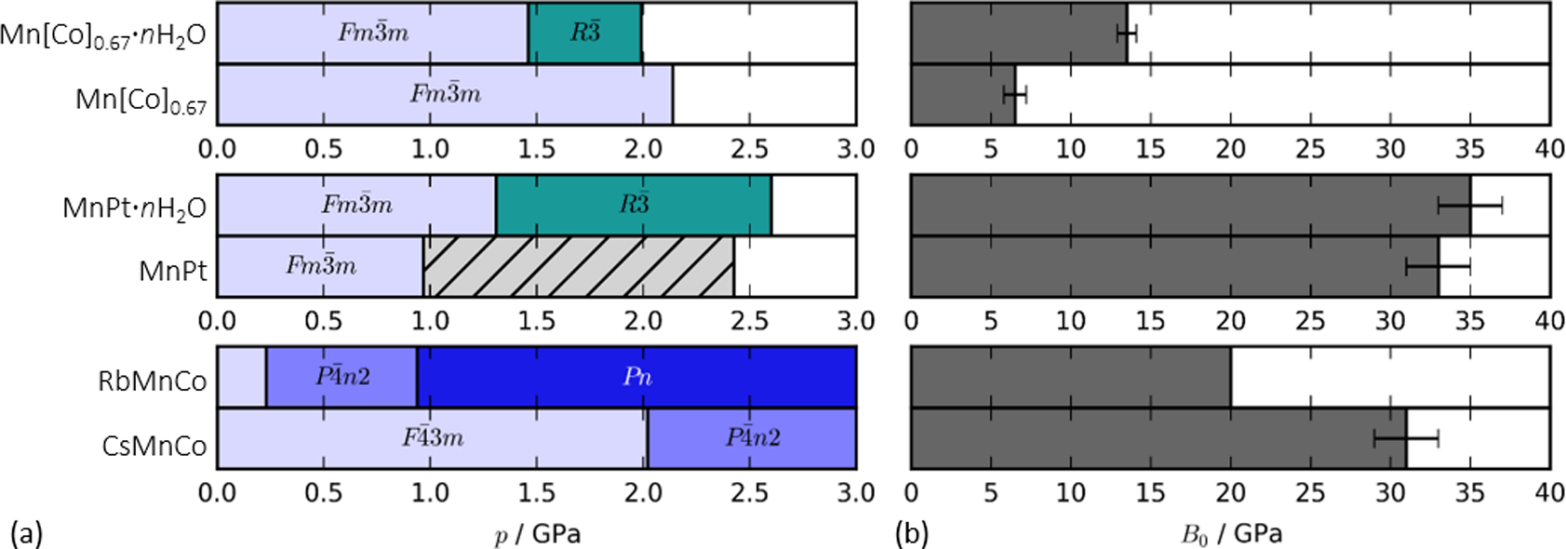
(a) The pressure–composition phase diagram of the Prussian blue analogues studied here. The hashed bar for MnPt corresponds to an unsolved phase. (b) The bulk moduli of the ambient phases as a function of composition. Note that the bulk modulus of RbMnCo is calculated based on two data points and should be interpreted with caution.

These results highlight the impact of compositional changes on the nonambient behavior of PBAs: defects soften the structure, water can modify the pressure-induced phase transitions, and the radius of the A-site alkali metal dictates the phase transition pressure [Figure 6]. These results will now be discussed in the context of related compounds.

The interplay between compression and defects noted here aligns with that observed in other coordination polymers. Despite being a challenging field of research,^36^ a few studies have been devoted to the role of defects on the compressibility of MOFs. To illustrate, removing 28% of the linkers in the archetypical UiO-66 reduces the bulk modulus by a factor of 2 and leads to amorphization at lower pressures.^35^ Likewise, metal vacancies (□) in [C(NH_2_)_3_]Fe_2/3_^III^□_1/3_(HCOO)_3_ lower the bulk modulus by 20% compared to the fully occupied [C(NH_2_)_3_]Mn^II^(HCOO)_3_ by increasing the strut compressibility.^77^ While in qualitative agreement, the defect-induced compressibility enhancement in these examples is substantially smaller than for the Prussian blue analogues—where removing ca. 25% of the transition metal clusters gives a 5-fold reduction of the bulk modulus. This emphasizes the high defect tolerance of the average PBA structure, which strongly impacts the softness of the material.

Changes in compressibility due to solvent inclusion—as observed for Mn[Co]_0.67_—is a general phenomenon for porous materials with potentially drastic effects. For example, the bulk modulus of the Cu-based MOF HKUST-1 nearly quadruples if a penetrating PTM is used, compared to a nonpenetrating one.^58^ Qualitatively similar results have been obtained for MOF-5^78^ and have been investigated in a range of zeolites where “*p*-induced overhydration” effects are observed when compressed in water-containing PTMs.^79^ An interesting avenue for further work would be to quantify the relationship between the hydration and bulk modulus. However, this requires accurate determination of water content and characterization of the hydration sites within the pores of the compound, a challenging task undoubtedly requiring a combination of experimental techniques. An important ramification of the solvent-dependent compressibility is that the mechanical properties of porous materials may depend on the ambient humidity, due to water adsorption from the atmosphere.

The role of water on the critical behavior of the stoichiometric MnPt is intriguing. While hydration-induced changes to the color and magnetic ordering temperature are reported in PBAs,^80,81^ these phenomena rely on the presence of vacancies. As water molecules coordinate to the open metal site, the ligand field is changed, which couples to the optical and magnetic properties.^80,81^ However, MnPt neither possesses open metal sites nor A-site cations to which the water molecules can coordinate, yet still features water-dependent phase transitions. It follows that the water, although often viewed as an inert interstitial, is not a spectator and can influence the phase stability. Further investigations would be worthwhile; however, as noted above, this is a nontrivial issue. The exact water content of an as-synthesized material likely depends on the synthesis conditions and ambient humidity, the latter of which is rarely controlled. Furthermore, the crystallographic study of water is complicated by its disordered nature, potentially requiring total neutron or X-ray scattering studies. Yet, some studies are emerging on the role of water in PBAs, which will largely benefit the community.^56,82,83^ In a wider context, our results also beg the question whether water may have a similar role in other coordination polymers and MOFs.

The hydration-dependent phase behavior of MnPt is reminiscent of the reductive intercalation of Na into FeFe(CN)_6_, exploited in positive electrodes for Na ion batteries.^4^ Insertion of Na^I^ induces a phase transition, but the symmetry of the Na-rich phase depends on the water content.^73^ Dehydrated Na_2_FeFe(CN)_6_ crystallizes in *R*3̅ (tilt system *a*^–^*a*^–^*a*^–^(14,62)), whereas the hydrated version adopts *P*2_1_/*n* symmetry (*a*^–^*a*^–^*c*^+^).^73^ Fully oxidized, cubic FeFe(CN)_6_ is structurally invariant to hydration.^84^ In effect, water acts as a transition modifier—it cannot induce phase transitions in cubic PBAs, but it dictates which transition occurs upon hydrostatic compression or Na^I^ inclusion. It is noteworthy that *dehydrated* Na_2_FeFe(CN)_6_ shows the same rhombohedral structure as *hydrated* MnPt·*n*H_2_O under compression.^23,73^ Thus, although not often recognized, water can play a fundamental role in the phase behavior of Prussian blue analogues.

Due to its polar symmetry, RbMnCo-III may in principle be a rare example of a PBA with ferroelectric properties. Polarity is extremely scarce in PBAs, although noncentrosymmetric space groups (*F*4̅3*m*) can arise for compounds with formula AMM′(CN)_6_, as the (R-point) order of the A-site cations breaks the inversion symmetry.^6,22,64^ However, these structures are still nonpolar—albeit piezoelectric—in the absence of further distortions. For RbMnCo-III, the pressure-induced *a*^–^*a*^–^*c*^+^ tilts reduce the symmetry to the polar space group *Pn*. By way of comparison, pressure-induced polarity is known for a handful of oxide perovskites, resulting from octahedral deformations upon compression.^85−87^ Group theory shows that out-of-phase tilts (“–”) are likely to give polar space groups when combined with the alternating A-site cation order.^45,88,89^ For example, *a*^–^*a*^–^*a*^–^ tilts of a cation-ordered AMM′(CN)_6_ compound would yield *R*3 symmetry. It may be possible to change the direction of the polarization by altering the direction of the tilt, and thereby generating a ferroelectric material, as in certain Ruddlesden–Popper oxides.^90,91^ This prospect of stimuli-induced polarity motivates further study into the tilt transitions of cation-ordered Prussian blue analogues.

The compositional tuning of phase transitions, as in AMnCo(CN)_6_, is a useful strategy for property optimization. In ABO_3_ perovskites, the relative radii of the A- and B-site metal cations is captured by the tolerance factor and this often correlates with the phase behavior and functionality.^92^ For example, both the metal–insulator and orthorhombic–rhombohedral phase transitions in LnNiO_3_ (Ln = lanthanoid) can be tuned by varying the size of Ln.^93^ Similar effects also occur in molecular compounds, e.g., PBAs with organic A-site cations, where the sizes of the M- and M′ metals stipulate the phase transition temperatures.^94,95^ In the case of AMnCo(CN)_6_, the large dependence on A suggests that the transition may be highly tunable over a wide pressure range by A-site substitution. This is particularly attractive in light of the polarity of the high-pressure phase of RbMnCo.

## Concluding Remarks

By means of variable-pressure diffraction, the roles of defects, hydration, and A-site cations on a range of Prussian blue analogues were investigated. Defects have a drastic effect on both the compressibility and phase transition behavior, with defective PBAs exhibiting remarkably low bulk moduli. No phase transitions occurred up to 2 GPa; instead, the crystallinity was reversibly reduced upon compression. It follows that defects greatly enhance the softness of PBAs, but also compromise the mechanical stability. As good stability is desirable for many applications,^24^ these results have implications for the development of devices based on defective PBAs, e.g., as gas sorbents.

Studies under pressure also revealed the curious and often overlooked impact of water on porous PBAs. Under ambient conditions, MnPt and Mn[Co]_0.67_ are not structurally altered by the presence of interstitial water molecules, yet the hydration state dictates the phase behavior on compression. This suggests that water modifies the accessible low-energy phonons, but this mechanism remains to be elucidated. The water-dependent phase behavior is mirrored by the phase transitions of FeFe(CN)_6_ upon Na intercalation, indicating that this may be a more general phenomenon.^73^ There is a clear need for further investigations into the water–framework interactions in a series of PBAs with quantified variable-hydration states.

The phase behavior of Prussian blue analogues with interstitial alkali metals is particularly significant for future materials design. RbMnCo exhibits the phase sequence *F*4̅3*m* → *P*4̅*n*2 → *Pn* below 1 GPa and seemingly remains in the polar *Pn* phase up to ca. 4 GPa. As a result, RbMnCo is one of the first Prussian blue analogues with a polar—and thus potentially ferroelectric—phase at industrially accessible pressures.^16^ Replacement of Rb with Cs shifts the *F*4̅3*m*–*P*4̅*n*2 transition to higher pressures, but no *Pn* phase was observed up to 3 GPa. If the *Pn* phase is accessible by higher pressures, it is possible to tune the pressure-induced polarity by A-site cation substitution. Studies of isostructural PBAs using high-pressure neutron diffraction will be an interesting avenue for further research.

Our results capture the key compositional factors at play in PBAs and can be generalized to other compositions. While this study has deliberately focused on qualitative effects to detangle a range of factors, the next step would be to quantify the interplay between, e.g., defects and the mechanism of compressibility. This work could furthermore facilitate better systematic understanding of additional factors that could affect the mechanical properties of PBAs for application, e.g., microstructure in nanocrystalline samples, or other behavior such as pressure-induced amorphization. This requires synthesizing samples with precisely defined compositions or crystallite size, which is a substantial challenge, but would greatly benefit the community. In addition to the relevance for PBAs, our results have a bearing on understanding the high-pressure behavior of a range of materials.

## Supporting Information Available

Diffraction patterns and fits to data, variable-pressure lattice parameters, mechanical building unit values, Birch–Murnaghan fits, crystallographic details for reported structures, supplementary discussion of pressure-induced amorphization (PDF)

## Accession Codes

CCDC 2048874–2048878 contain the supplementary crystallographic data for this paper. These data can be obtained free of charge via www.ccdc.cam.ac.uk/data_request/cif, or by emailing data_request@ccdc.cam.ac.uk, or by contacting The Cambridge Crystallographic Data Centre, 12 Union Road, Cambridge CB2 1EZ, UK; fax: +44 1223 336033.
