# Supplementary material for: Probing the Influence of Defects, Hydration, and Composition on Prussian Blue Analogues with Pressure
Source: J Am Chem Soc. 2021 Feb 25;143(9):3544–54. doi: 10.1021/jacs.0c13181 (PMC8028041; doi:10.1021/jacs.0c13181)
Supplement: Supplementary file 1 — ja0c13181_si_001.pdf [file ja0c13181_si_001.pdf]

Probing the Influence of Defects, Hydration and Composition on Prussian Blue Analogues with Pressure
SUPPORTING INFORMATION

Hanna L. B. Boström,[a,b,c*] Ines E. Collings,[d] Dominik Daisenberger,[e]
Christopher J. Ridley,[f] Nicholas P. Funnell,[f] and Andrew B. Cairns[g,h*]

[a] Max Planck Institute for Solid State Research, Heisenbergstraße 1, D-70569 Stuttgart, Germany.
[b] Department of Inorganic Chemistry, Ångström Laboratory, Uppsala University, Box 538, SE-751 21 Uppsala, Sweden.
[c] Department of Chemistry, University of Oxford, Inorganic Chemistry Laboratory South Parks Road, Oxford OX1 3QR, U.K.
[d] Centre for X-ray Analytics, EMPA - Swiss Federal Laboratories for Materials Science and Technology, Überlandstrasse 129, 8600 Dübendorf, Switzerland.
[e] Diamond Light Source Ltd., Harwell Campus, Didcot OX11 0DE, U.K.
[f] ISIS Neutron and Muon Source, Rutherford Appleton Laboratory, Harwell Campus, Didcot OX11 0QX, U.K.
[g] Department of Materials, Imperial College London, Royal School of Mines, Exhibition Road, SW7 2AZ, London, U.K.
[h] London Centre for Nanotechnology, Imperial College London, SW7 2AZ, London, U.K.

*To whom correspondence should be addressed;
E-mail: h.bostroem@fkf.mpg.de, a.cairns@imperial.ac.uk

Contents

1 Variable-pressure diffraction fits **3**

2 Variable-pressure lattice parameters **14**

3 Mechanical building unit (XBU) values **19**

4 Birch-Murnaghan fits **19**

5 The equation of state for CsMnCo **24**

6 Crystallographic details of new phases **27**

7 Pressure-induced amorphisation (PIA) in MnPt$\cdot n$D$_2$O and Mn[Co]$_{0.67}\cdot n$D$_2$O **35**

8 References **38**

1 Variable-pressure diffraction fits

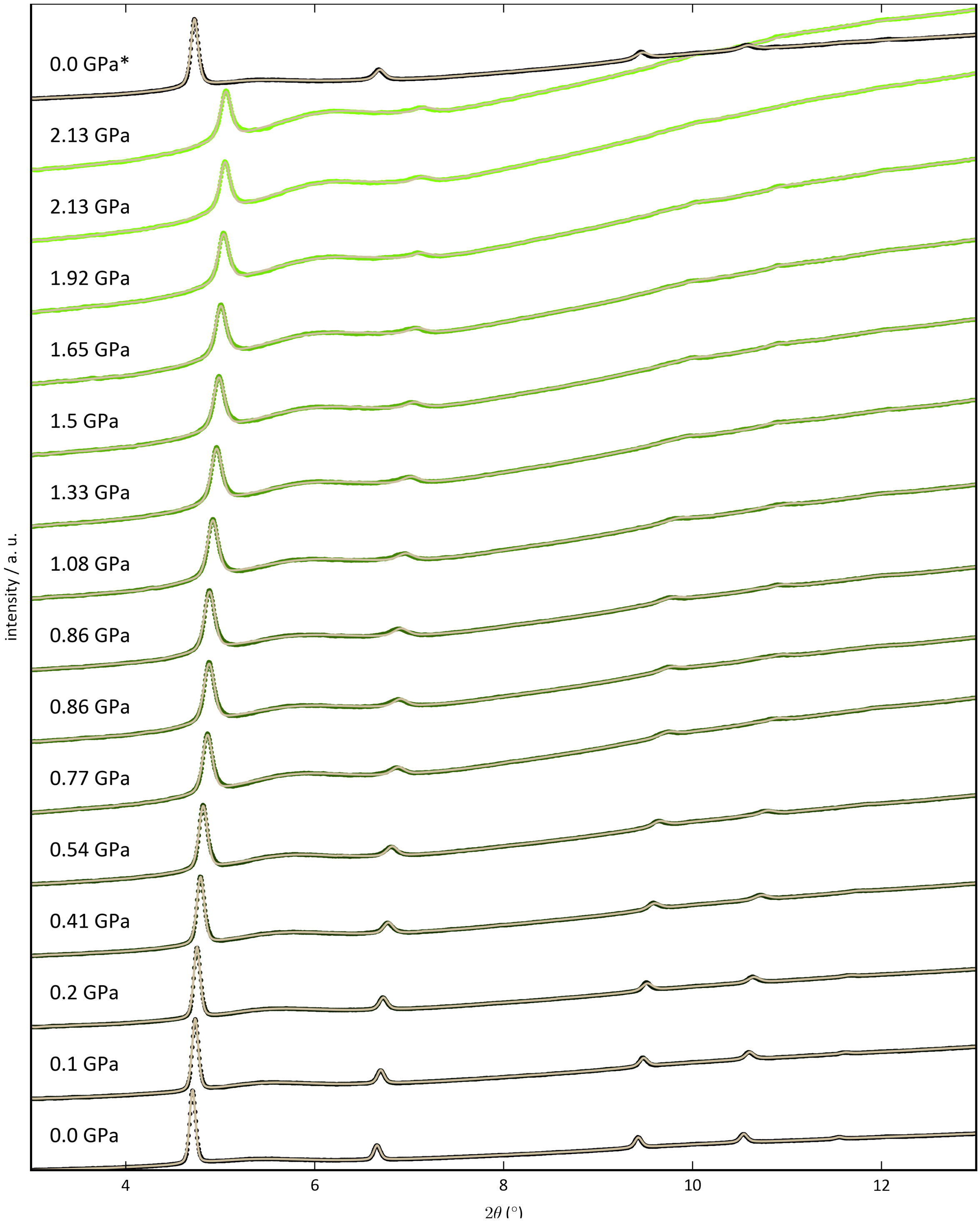

Figure S1: The high-pressure X-ray diffraction patterns of dehydrated Mn[Co]$_{0.67}$ with observed data shown in black/green and calculated from Pawley refinement in beige. Data collected on decompression are denoted by an asterisk. $\lambda = 0.424112$ Å

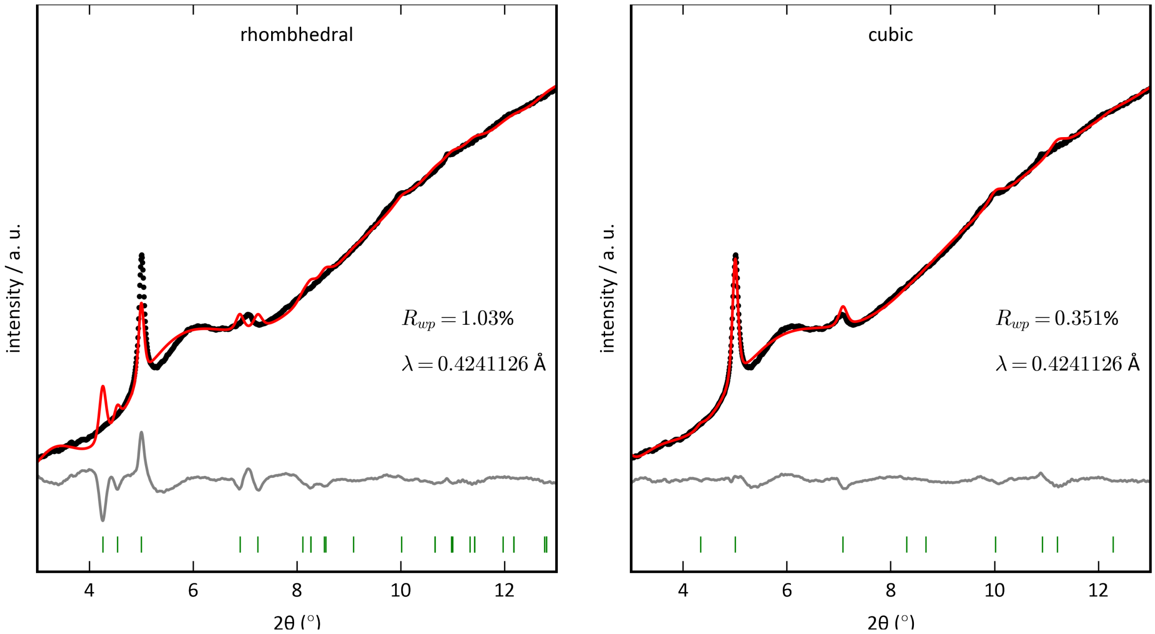

Figure S2: The Rietveld fit of Mn[Co]$_{0.67}$ at 2.14 GPa to the rhombohedral $R\bar{3}$ phase, found for Mn[Co(CN)$_6$]$_{2/3}$ · xH$_2$O (Ref. 1), compared to the cubic $Fm\bar{3}m$ phase. The experimental data shown in black, the fit in red, residuals in grey and allowed reflections indicated by vertical bars.

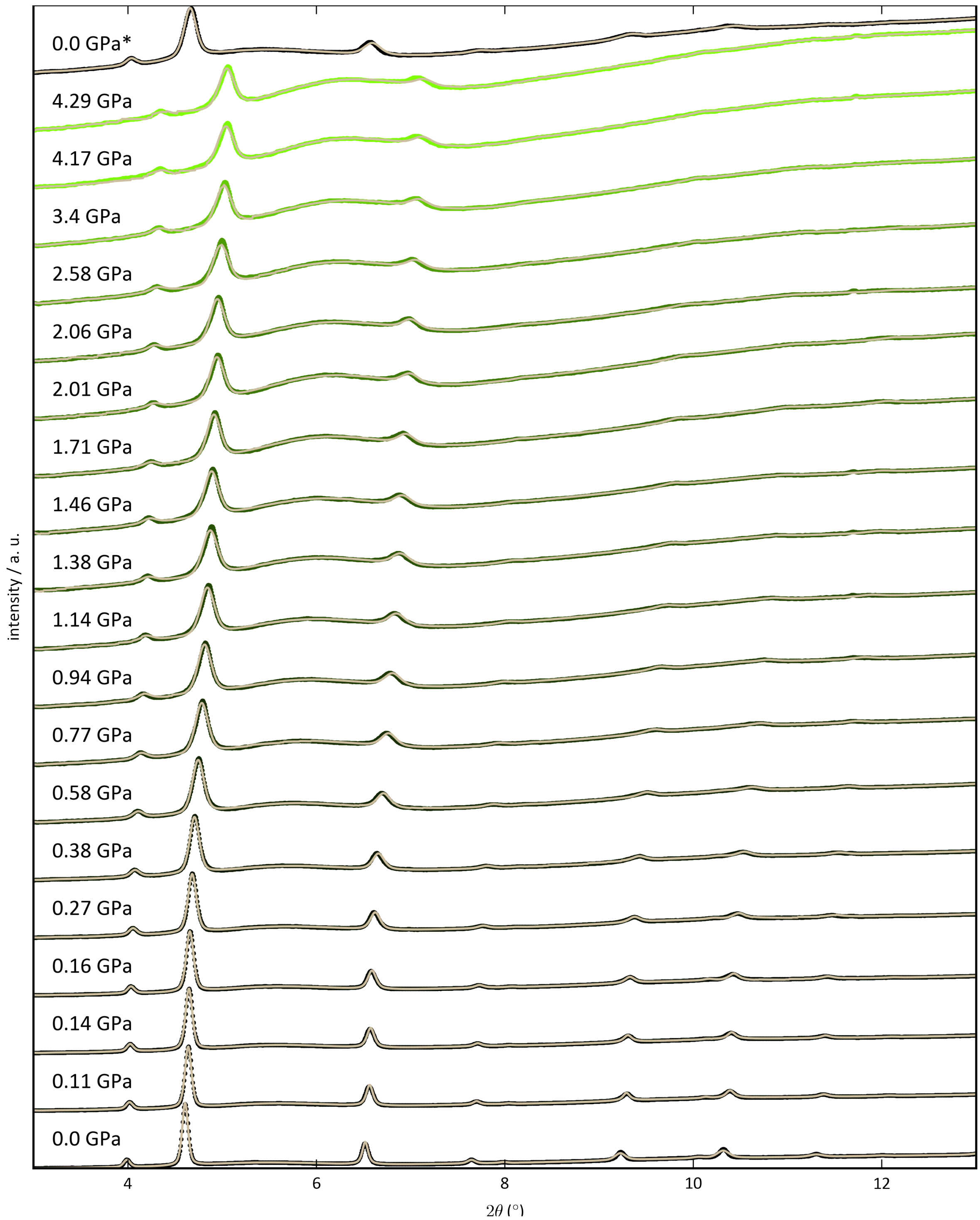

Figure S3: The high-pressure X-ray diffraction patterns of dehydrated $Cd[Co]_{0.67}$ with observed data shown in black/green and calculated from Pawley refinement in beige. Data collected on decompression are denoted by an asterisk. $\lambda = 0.424112$ Å

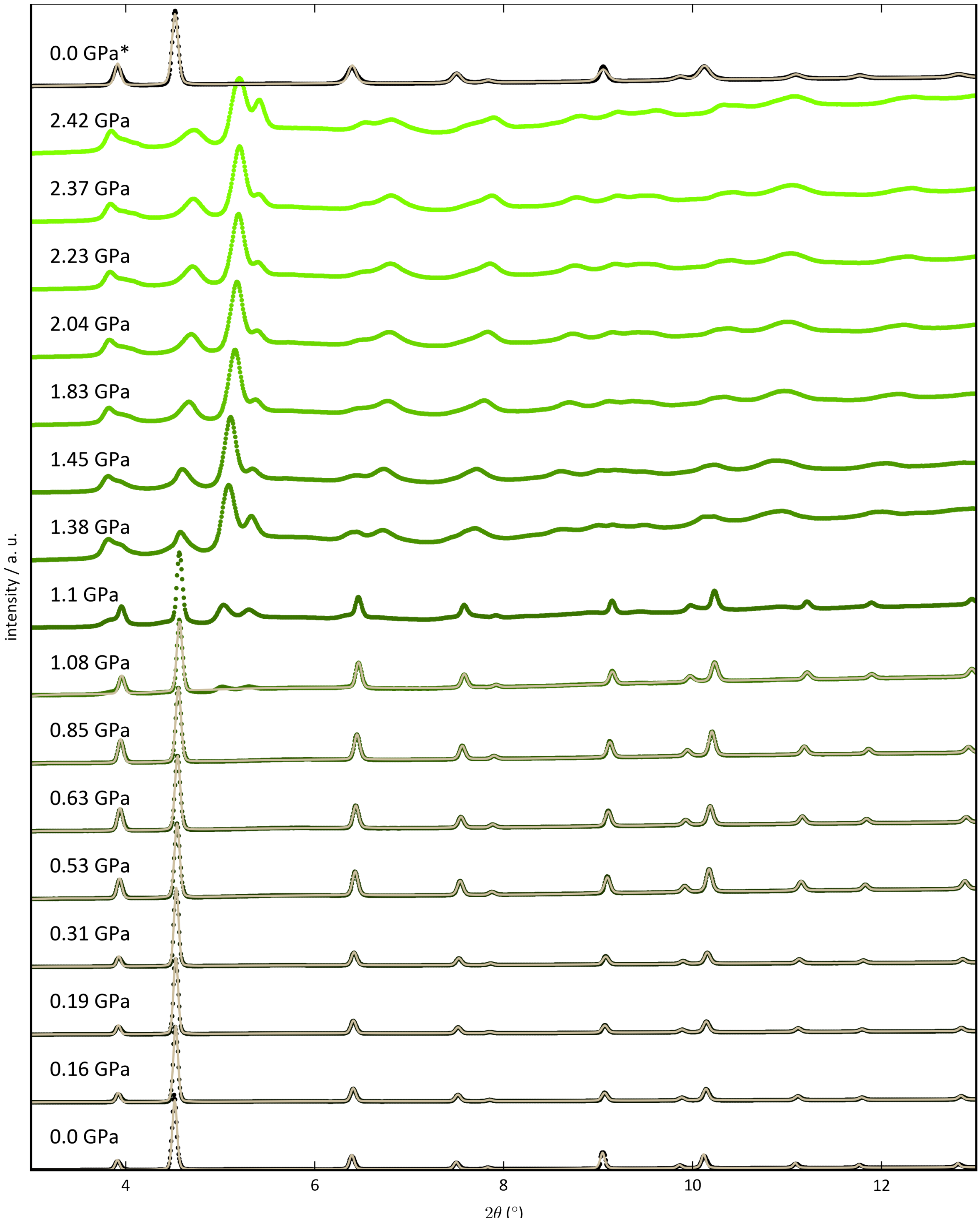

Figure S4: The high-pressure X-ray diffraction patterns of dehydrated MnPt with observed data shown in black/green and calculated from Pawley refinement in beige. Data collected on decompression are denoted by an asterisk. $\lambda = 0.424112\,\text{Å}$

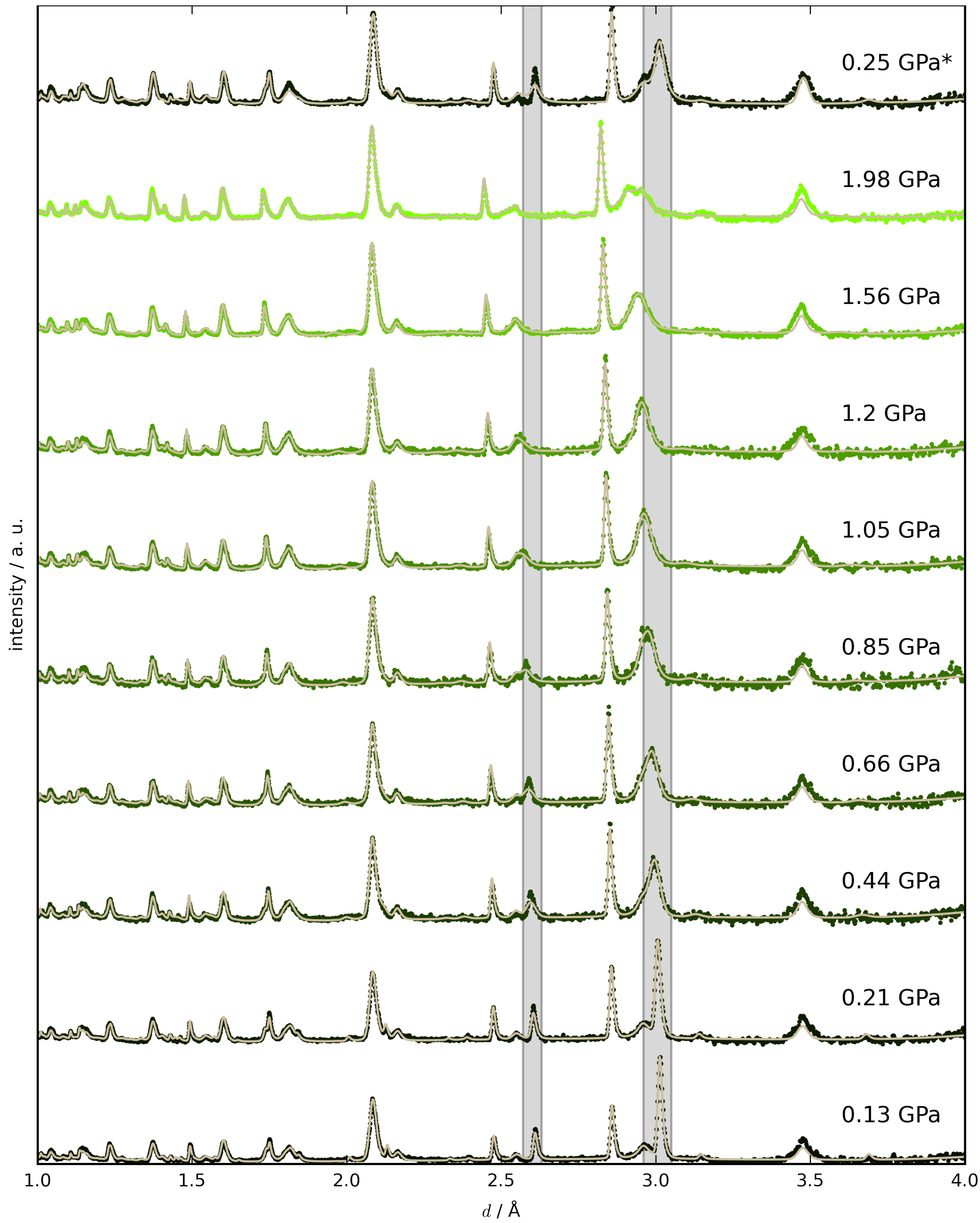

Figure S5: The high-pressure neutron diffraction patterns of hydrated $Mn[Co]_{0.67} \cdot nD_2O$ with observed data shown in black/green and calculated from Rietveld refinement in beige. Data collected on decompression are denoted by an asterisk. Key reflections from the sample are highlighted in grey.

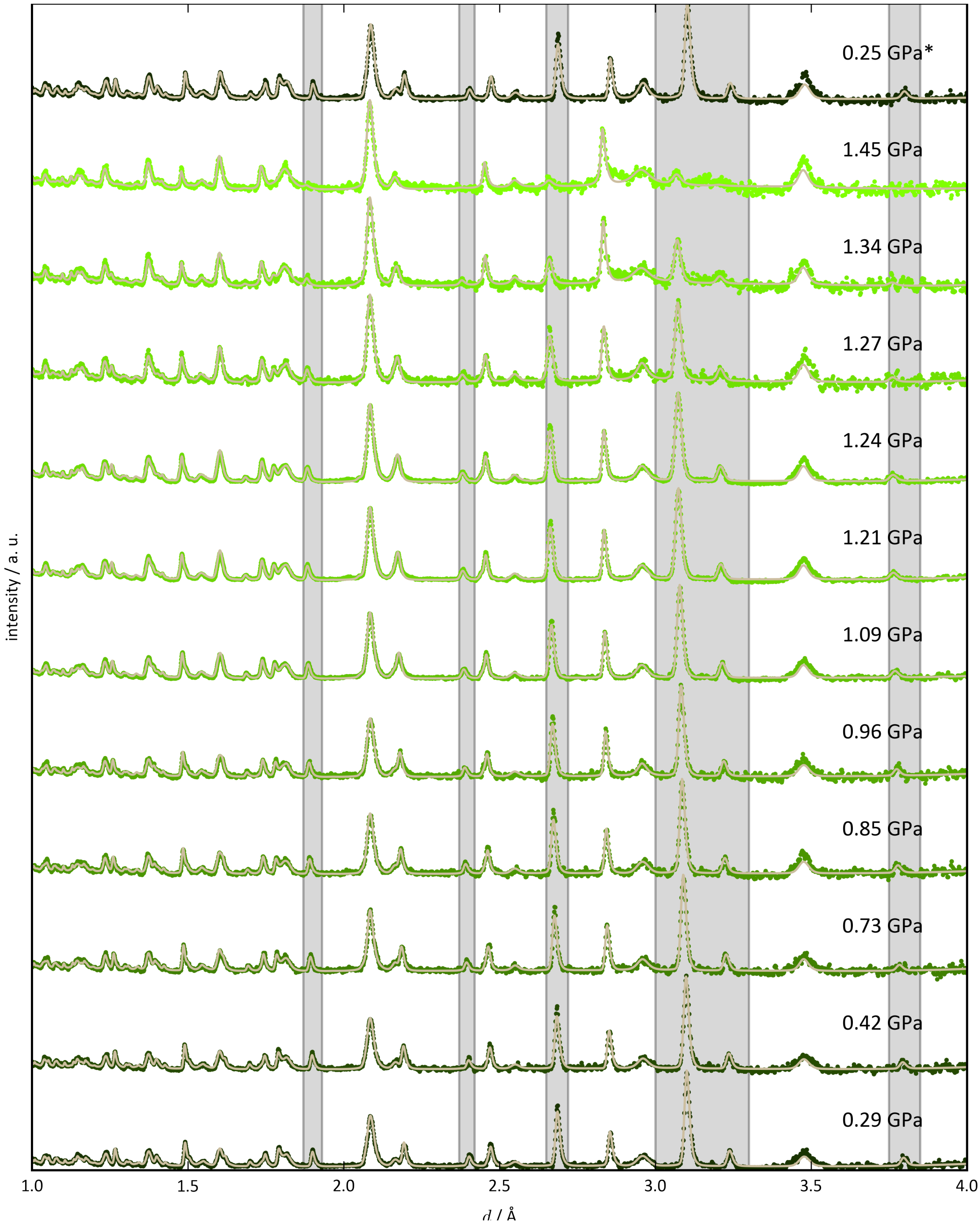

Figure S6: The high-pressure neutron diffraction patterns of hydrated MnPt·$n$$D_2O$ with observed data shown in black/green and calculated from Rietveld refinement in beige. Data collected on decompression are denoted by an asterisk. Key reflections from the sample are highlighted in grey.

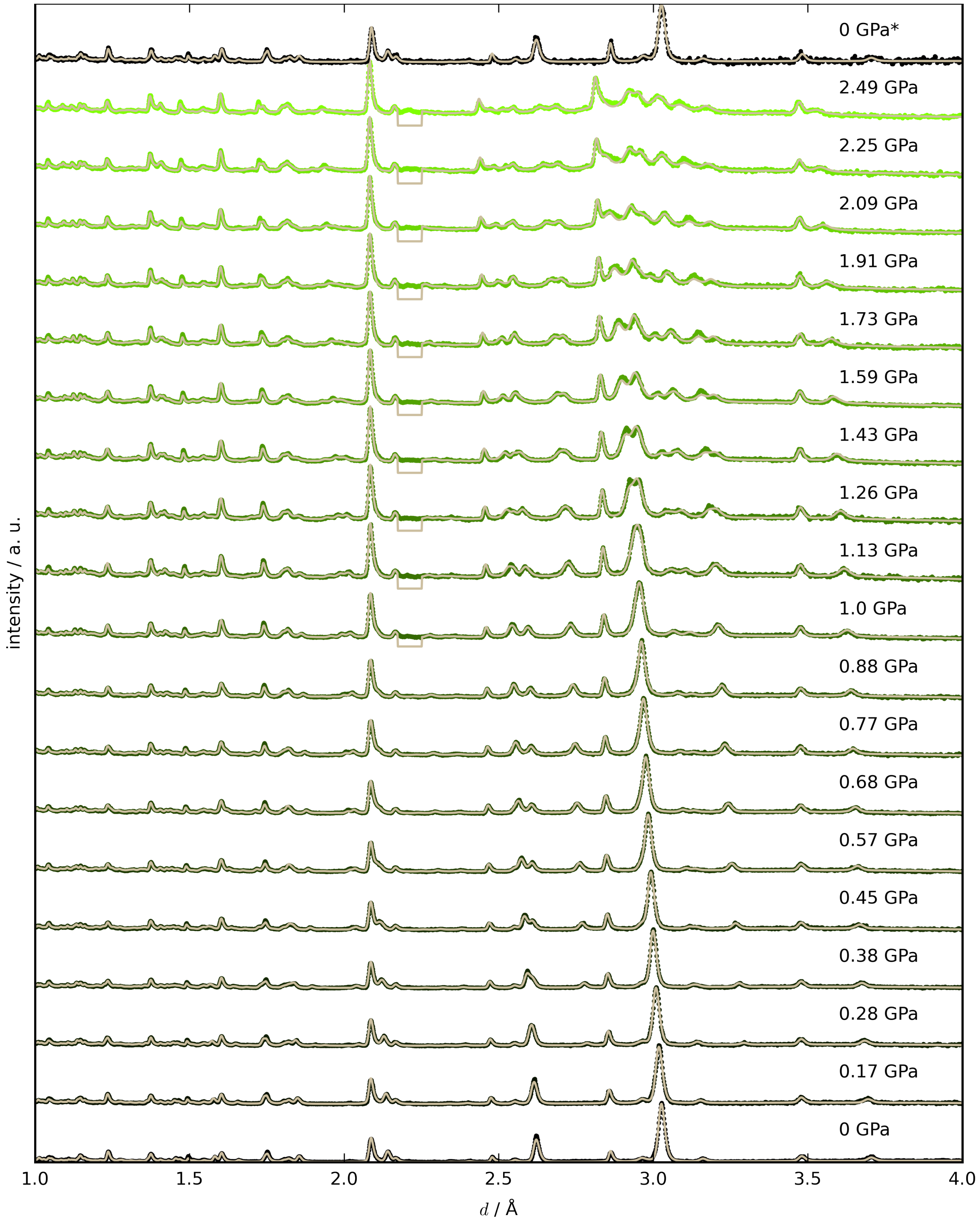

Figure S7: The high-pressure neutron diffraction patterns of RbMnCo with observed data shown in black/green and calculated from Rietveld refinement in beige. Data collected on decompression are denoted by an asterisk. The region at 2.2 Å contains an additional reflection from the anvil material and was excluded.

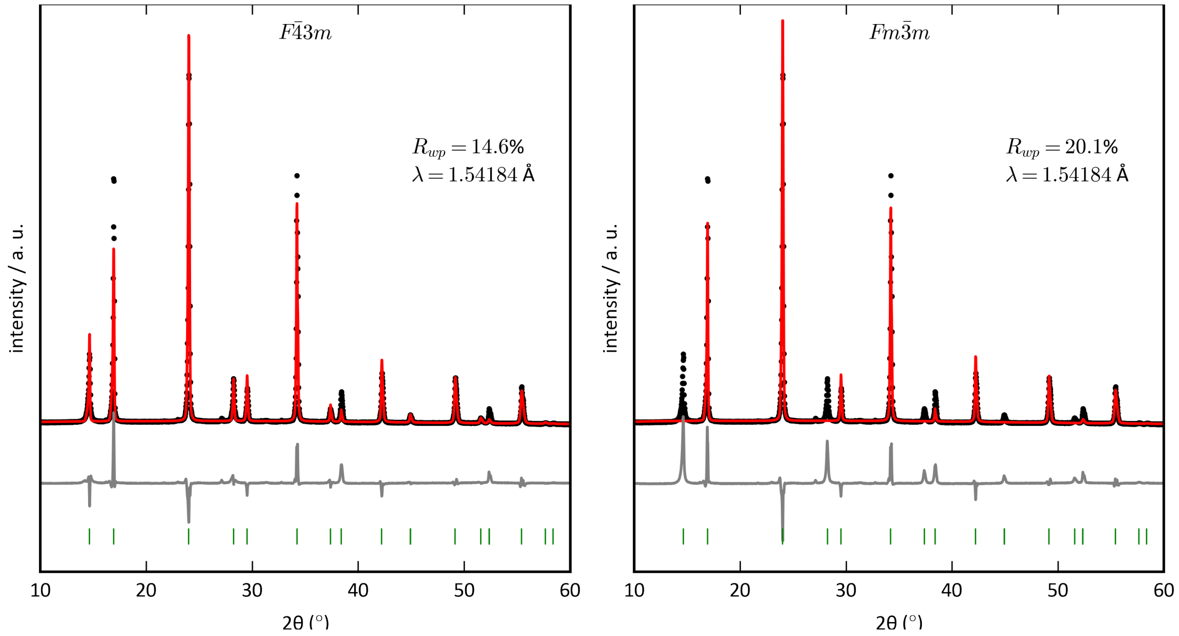

Figure S8: The Rietveld fit of RbMnCo in noncentrosymmetric $F\bar{4}3m$ and centrosymmetric $Fm\bar{3}m$ to XRD data. The experimental data are shown in black, the fit in red, the residuals in grey and allowed reflections indicated by vertical bars.

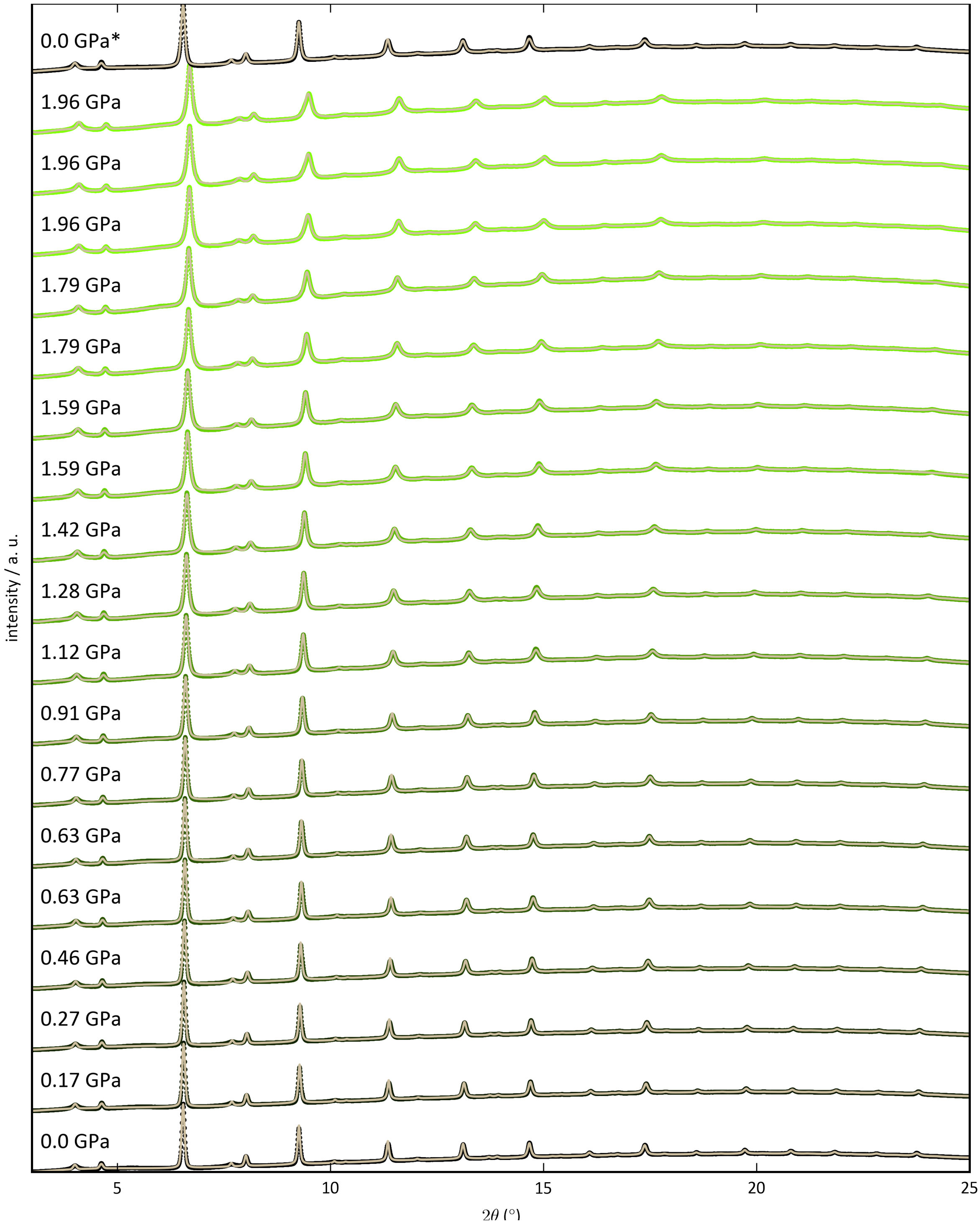

Figure S9: The high-pressure X-ray diffraction patterns of the ambient phase of CsMnCo with observed data shown in black/green and calculated from Pawley refinement in beige. Data collected on decompression are denoted by an asterisk. $\lambda = 0.424112$ Å

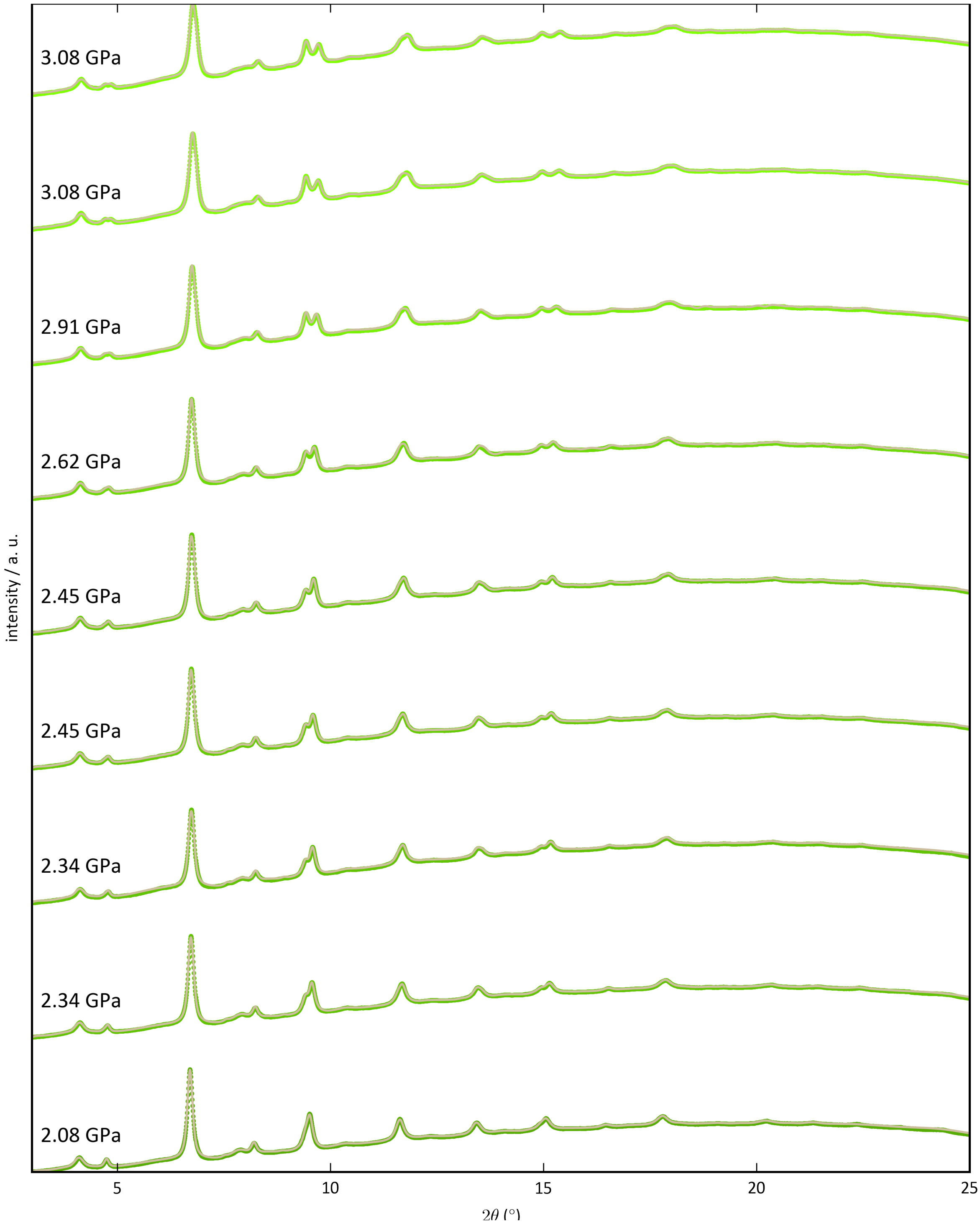

Figure S10: The high-pressure X-ray diffraction patterns of the high-pressure phase of CsMnCo with observed data shown in black/green and calculated from Pawley refinement in beige. $\lambda = 0.424112$ Å

2 Variable-pressure lattice parameters

Table S1: Variable-pressure lattice parameters of dehydrated Mn[Co]$_{0.67}$ measured at I15. Space group is $Fm\bar{3}m$. Estimated uncertainty is ± 0.1 GPa at all pressures.

p / GPa	a / Å
0	10.3218(2)
0.095	10.2617(2)
0.205	10.2202(2)
0.41	10.1424(3)
0.535	10.0856(2)
0.77	9.9897(3)
0.865	9.9577(5)
0.865	9.9467(4)
1.085	9.8793(7)
1.335	9.7973(4)
1.5	9.7468(7)
1.65	9.7086(7)
1.915	9.6524(8)
2.135	9.6240(9)
2.135	9.6069(9)

Table S2: Variable-pressure lattice parameters of dehydrated Cd[Co]$_{0.67}$ measured at I15. Space group is $Fm\bar{3}m$. Estimated uncertainty is ± 0.1 GPa at all pressures.

p / GPa	a / Å
0	10.5468(2)
0.11	10.4724(2)
0.14	10.4556(2)
0.16	10.4327(2)
0.27	10.3783(3)
0.38	10.3247(3)
0.58	10.2394(7)
0.765	10.158(9)
0.945	10.09604)
1.140	10.0345(5)
1.375	9.9736(7)
1.46	9.9448(7)
1.705	9.8936(4)
2.01	9.8318(7)
2.055	9.8215(7)

Table S3: Variable-pressure lattice parameters of dehydrated MnPt measured at I15. Space group is $Fm\bar{3}m$. Estimated uncertainty is ± 0.1 GPa at all pressures.

p / GPa	a / Å
0	10.7532(2)
0.16	10.7281(12)
0.19	10.72501(10)
0.31	10.71344(11)
0.53	10.69324(8)
0.63	10.68155(8)
0.85	10.66173(8)

Table S4: Variable-pressure lattice parameters of hydrated $Mn[Co]_{0.67}\cdot nD_2O$ measured at PEARL. Space group is $Fm\bar{3}m$.

p / GPa	a / Å
0.128(5)	10.4287(2)
0.211(4)	10.4050(3)
0.439(5)	10.3651(11)
0.657(6)	10.3363(14)
0.849(7)	10.302(2)
1.050(7)	10.260(2)
1.200(8)	10.229(3)
1.557(11)	10.150(4)
1.98(2)	10.063(4)

Table S5: Variable-pressure lattice parameters of hydrated $MnPt\cdot nD_2O$ measured at PEARL. Space group is $Fm\bar{3}m$.

p / GPa	a / Å
0.289(6)	10.7333(2)
0.422(7)	10.7254(2)
0.728(7)	10.6948(3)
0.846(8)	10.6814(3)
0.956(8)	10.6703(3)
1.095(6)	10.6543(2)
1.210(7)	10.6387(2)
1.241(7)	10.6355(3)
1.271(10)	10.6329(5)
1.344(9)	10.6266(9)
1.448(11)	10.618(4)
0.249(7)	10.7397(3)

Table S6: Variable-pressure lattice parameters of dehydrated RbMnCo measured at PEARL.

p / GPa	space group	a / Å	b / Å	c / Å	β (°)
0	$F\bar{4}3m$	10.47265(11)			90
0.172(5)	$F\bar{4}3m$	10.44379(13)			90
0.281(5)	$P\bar{4}n2$	7.3567(4)		10.4263(13)	90
0.376(5)	$P\bar{4}n2$	7.3225(2)		10.4275(5)	90
0.449(5)	$P\bar{4}n2$	7.2971(2)		10.4227(7)	90
0.567(5)	$P\bar{4}n2$	7.2683(2)		10.4163(6)	90
0.678(5)	$P\bar{4}n2$	7.2429(2)		10.4107(7)	90
0.773(5)	$P\bar{4}n2$	7.2190(3)		10.4038(9)	90
0.881(6)	$P\bar{4}n2$	7.1972(3)		10.3942(9)	90
1.002(6)	Pn	7.1580(15)	7.1877(14)	10.3610(9)	89.71(2)
1.126(7)	Pn	7.1234(11)	7.1836(19)	10.3327(13)	89.93(3)
1.256(7)	Pn	7.0855(9)	7.1789(8)	10.2949(15)	90.01(5)
1.429(8)	Pn	7.0491(9)	7.1742(9)	10.262(2)	90.00(6)
1.592(9)	Pn	7.0168(10)	7.1715(9)	10.224(2)	90.02(6)
1.731(11)	Pn	6.9925(11)	7.1720(10)	10.195(2)	90.02(7)
1.907(12)	Pn	6.9540(12)	7.1612(12)	10.164(3)	90.03(7)
2.095(14)	Pn	6.9125(13)	7.1542(12)	10.127(3)	89.98(7)
2.254(15)	Pn	6.876(2)	7.1501(15)	10.103(3)	90.03(8)
2.49(2)	Pn	6.835(2)	7.149(2)	10.057(3)	89.87(7)

Table S7: Variable-pressure lattice parameters of dehydrated $CsMnCo(CN)_6$ measured at I15. Estimated uncertainty is ± 0.1 GPa at all pressures.

p / GPa	space group	a / Å	c / Å
0	$F\bar{4}3m$	10.50335(09)	
0.18	$F\bar{4}3m$	10.48531(10)	
0.27	$F\bar{4}3m$	10.47413(10)	
0.46	$F\bar{4}3m$	10.45583(12)	
0.63	$F\bar{4}3m$	10.43828(10)	
0.63	$F\bar{4}3m$	10.43688(13)	
0.77	$F\bar{4}3m$	10.42554(13)	
0.91	$F\bar{4}3m$	10.4114(2)	
1.13	$F\bar{4}3m$	10.3921(2)	
1.29	$F\bar{4}3m$	10.38012(10)	
1.43	$F\bar{4}3m$	10.3622(2)	
1.60	$F\bar{4}3m$	10.3390(2)	
1.60	$F\bar{4}3m$	10.3327(2)	
1.79	$F\bar{4}3m$	10.3042(2)	
1.79	$F\bar{4}3m$	10.2959(2)	
1.96	$F\bar{4}3m$	10.2734(2)	
1.96	$F\bar{4}3m$	10.2664(2)	
1.96	$F\bar{4}3m$	10.2640(2)	
2.08	$P\bar{4}n2$	7.2286(4)	10.334(2)
2.34	$P\bar{4}n2$	7.1895(3)	10.3378(8)
2.34	$P\bar{4}n2$	7.1792(3)	10.3383(9)
2.45	$P\bar{4}n2$	7.1717(3)	10.3349(7)
2.45	$P\bar{4}n2$	7.1598(3)	10.3352(7)
2.62	$P\bar{4}n2$	7.1487(6)	10.3153(12)
2.91	$P\bar{4}n2$	7.1124(7)	10.3168(9)
3.08	$P\bar{4}n2$	7.0856(5)	10.3177(6)
3.08	$P\bar{4}n2$	7.0855(10)	10.3156(11)

3 Mechanical building unit (XBU) values

Table S8: Formulae relating r_{ab}, r_c, and ϕ to the lattice parameters

	cubic	tetragonal	monoclinic
r_{ab}	a	$\sqrt{2}\,a$	$\sqrt{a^2+b^2}$
r_c	a	c	c
ϕ	90°	90°	$2\arctan\left(\frac{a}{b}\right)$

Table S9: Principal axes compressibilities of the low-symmetry phases calculated by PASCal.[S2]

	K_a / TPa^{-1}	K_b / TPa^{-1}	K_c / TPa^{-1}
RbMnCo-II	30.8(9)		6.6(4)
RbMnCo-III	26(1)	4.0(7)	20.2(9)
CsMnCo-II	18(2)		3(3)

4 Birch-Murnaghan fits

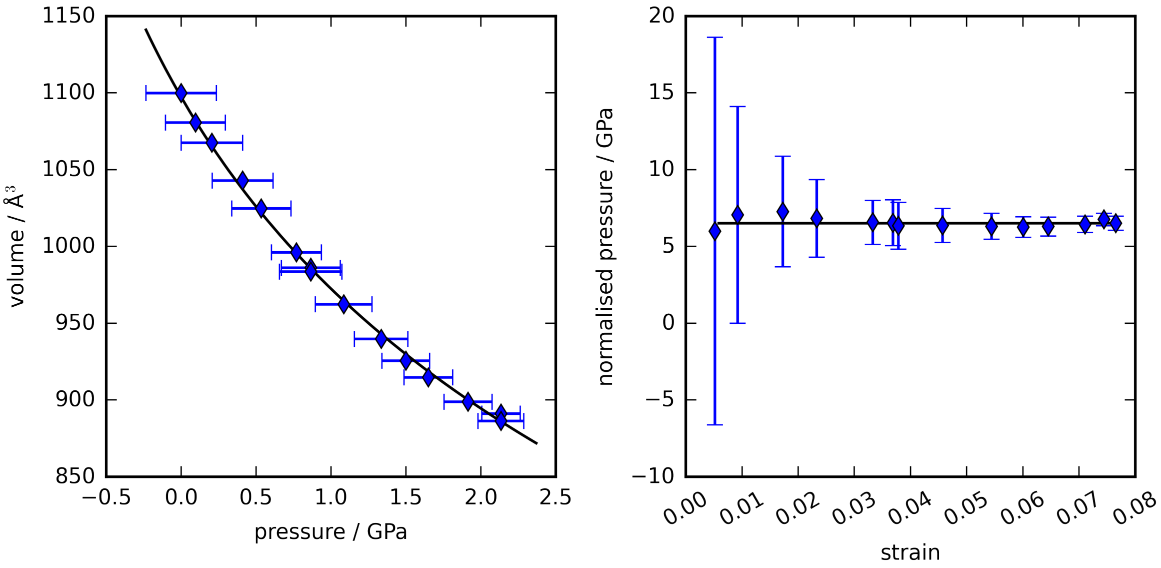

Figure S11: (left) Fits to experimental unit cell volumes using second-order Birch-Murnaghan equation of states and (right) the normalised pressure *vs.* Eulerian strain for dehydrated Mn[Co]$_{0.67}$.

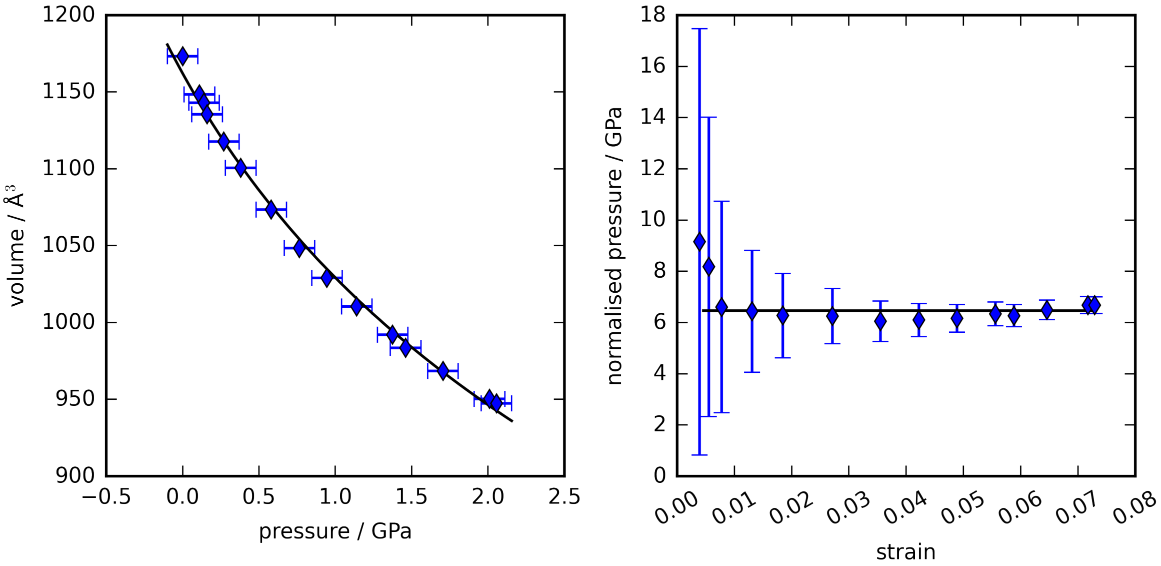

Figure S12: (left) Fits to experimental unit cell volumes using second-order Birch-Murnaghan equation of states and (right) the normalised pressure *vs.* Eulerian strain for dehydrated Cd[Co]$_{0.67}$.

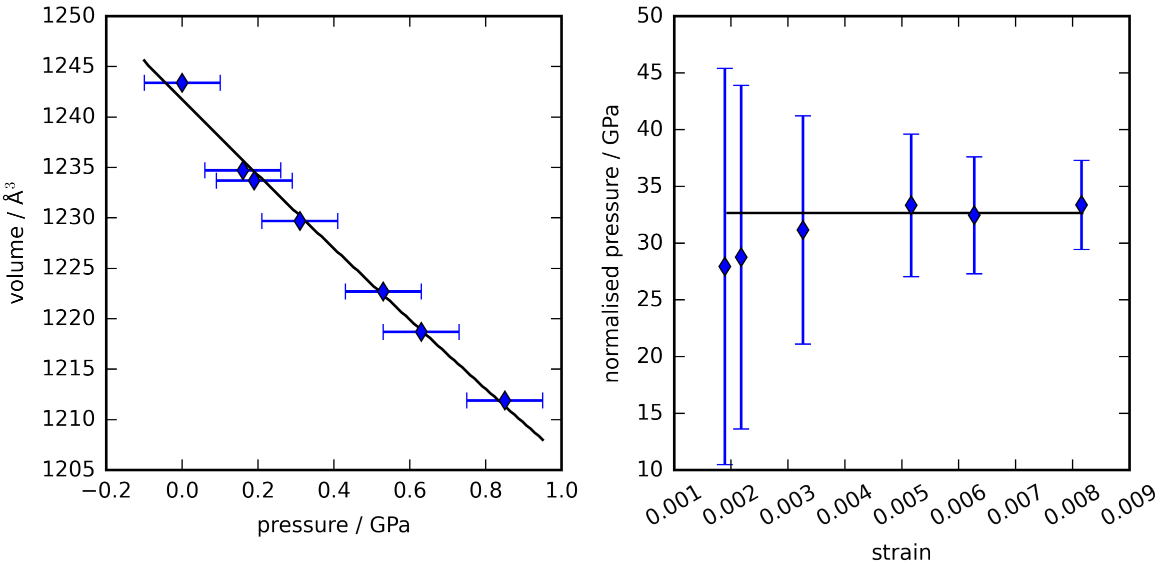

Figure S13: (left) Fits to experimental unit cell volumes using second-order Birch-Murnaghan equation of states and (right) the normalised pressure *vs.* Eulerian strain for dehydrated MnPt.

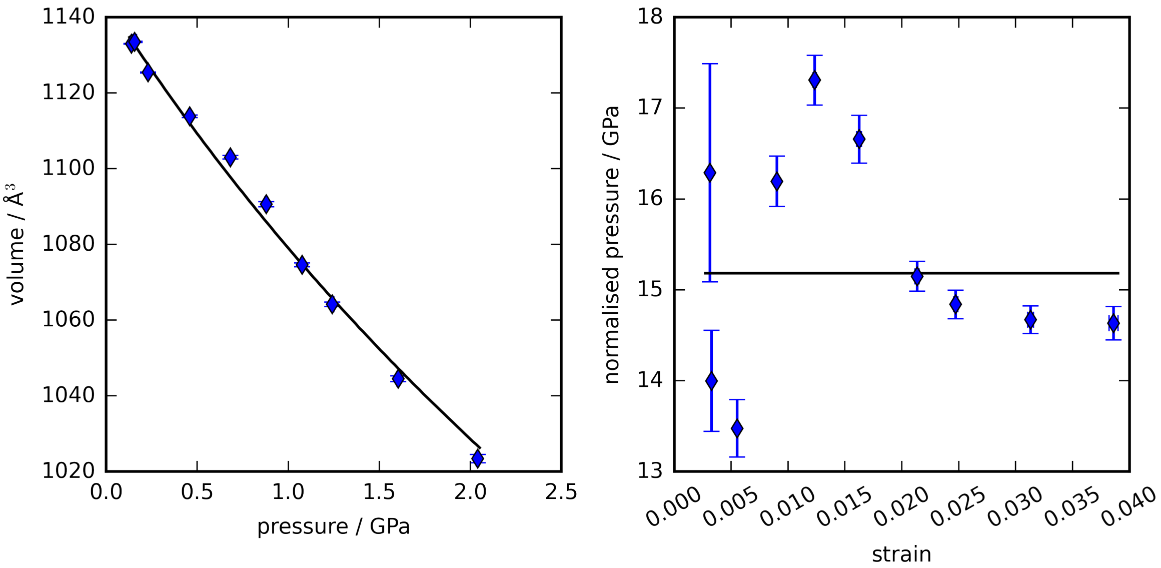

Figure S14: (left) Fits to experimental unit cell volumes using second-order Birch-Murnaghan equation of states and (right) the normalised pressure *vs.* Eulerian strain for hydrated Mn[Co]$_{0.67}$·nD$_2$O.

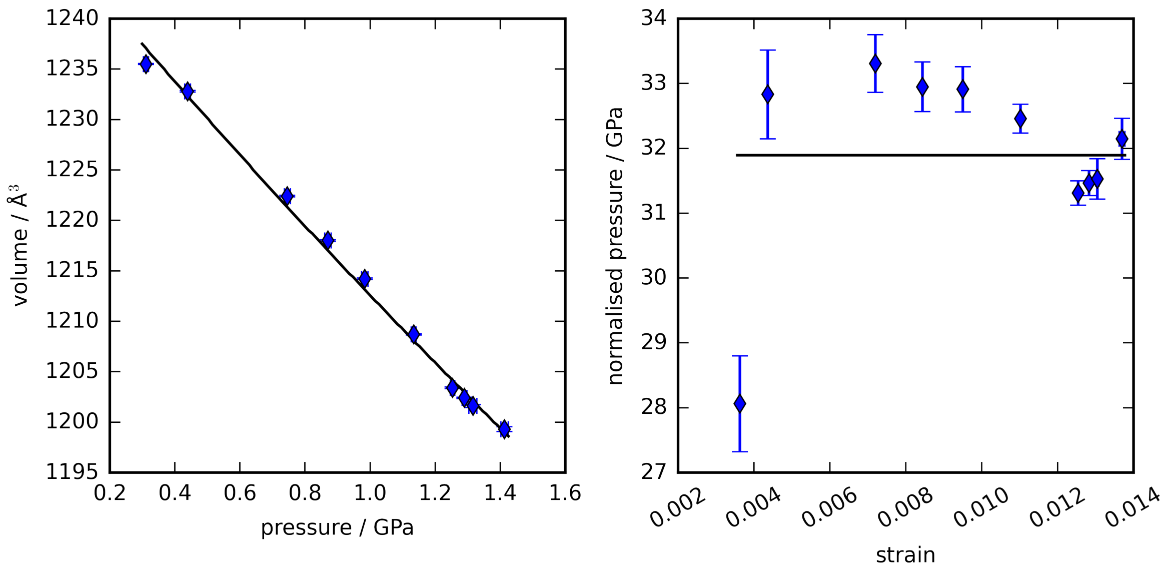

Figure S15: (left) Fits to experimental unit cell volumes using second-order Birch-Murnaghan equation of states and (right) the normalised pressure *vs.* Eulerian strain for hydrated MnPt·nD$_2$O.

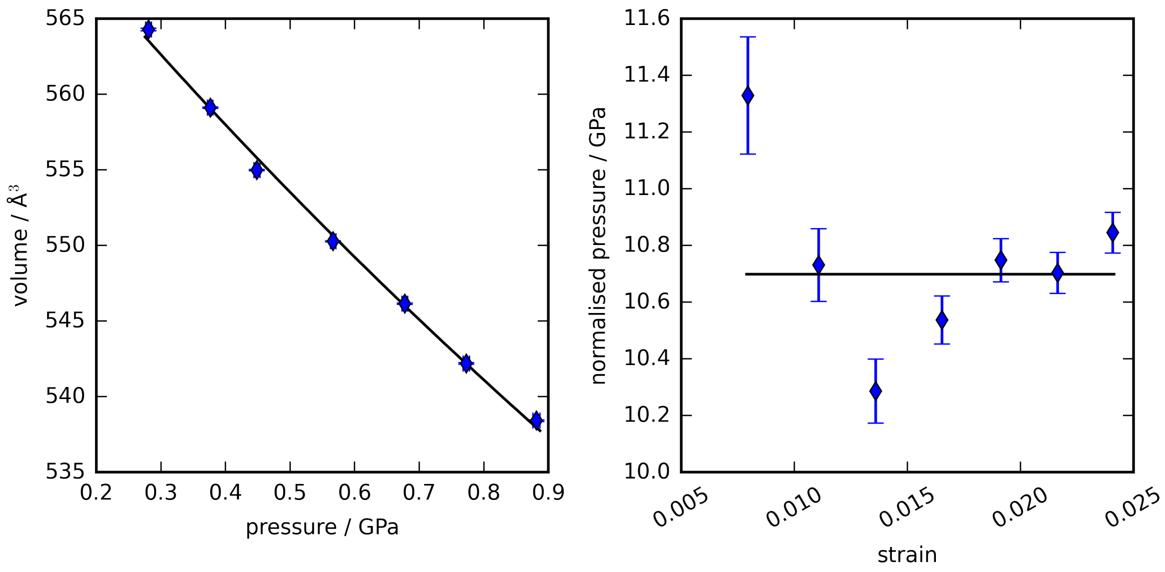

Figure S16: (left) Fits to experimental unit cell volumes using second-order Birch-Murnaghan equation of states and (right) the normalised pressure *vs.* Eulerian strain for the tetragonal phase RbMnCo-II.

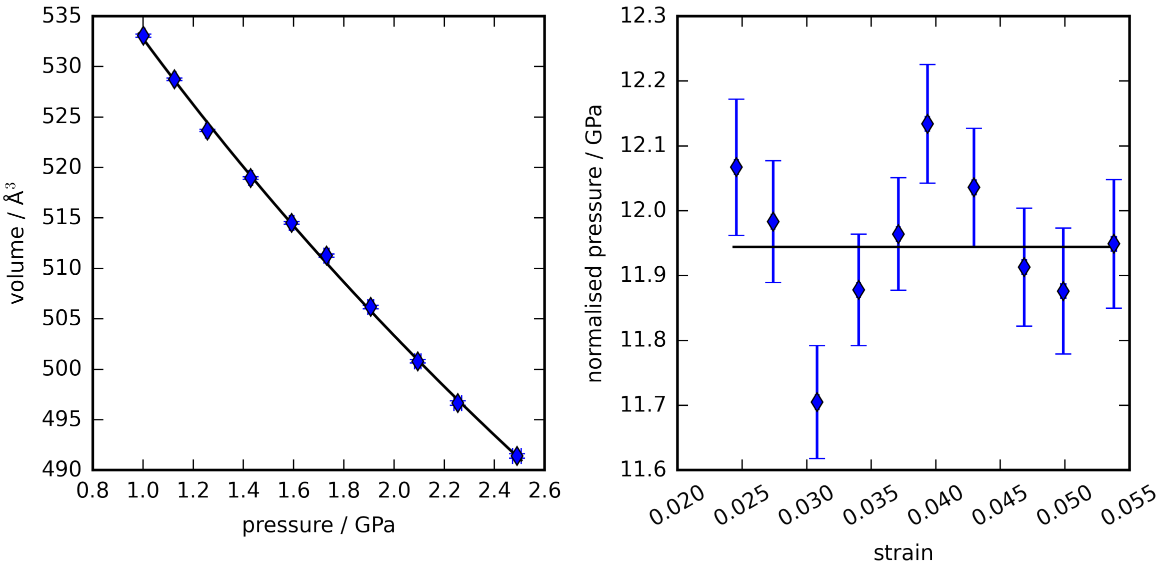

Figure S17: (left) Fits to experimental unit cell volumes using second-order Birch-Murnaghan equation of states and (right) the normalised pressure *vs.* Eulerian strain for the monoclinic phase RbMnCo-III.

5 The equation of state for CsMnCo

A second-order Birch-Murnaghan[S3–5] fit of the ambient phase of CsMnCo in the range 0–1.6 GPa gives $B_0 = 31(2)$ GPa [Fig. S18]. Conversely, employing a third-order fit in the range 0–2 GPa yields $B_0 = 42(3)$ GPa and $B' = -7(2)$, where $B' = dB_0/dp$. The negative value of B' indicates pressure-induced softening, a feature sometimes observed before a phase transition and in negative thermal expansion (NTE) materials.[S6–9] The third-order fit suggests that CsMnCo is initially less compressible than the dehydrated A-site-deficient MnPt ($B = 33(2)$ GPa), but then rapidly softens upon compression. In contrast, the second-order fit suggests these two systems have very similar compressibilities prior to the onset of any phase transition. Caution should be taken when comparing bulk moduli values obtained from different orders of fit; the gradual nature of the phase transition gives rise to uncertainty in the true transition pressure and therefore if the drop volume in this region is preceding, or a result of, the phase transition. Nonetheless, both approaches give acceptable fits in the pressure range stated [Fig. S18].

CsMnCo-II was well fitted by a second-order Birch–Murnaghan equation of state [Fig. S19].

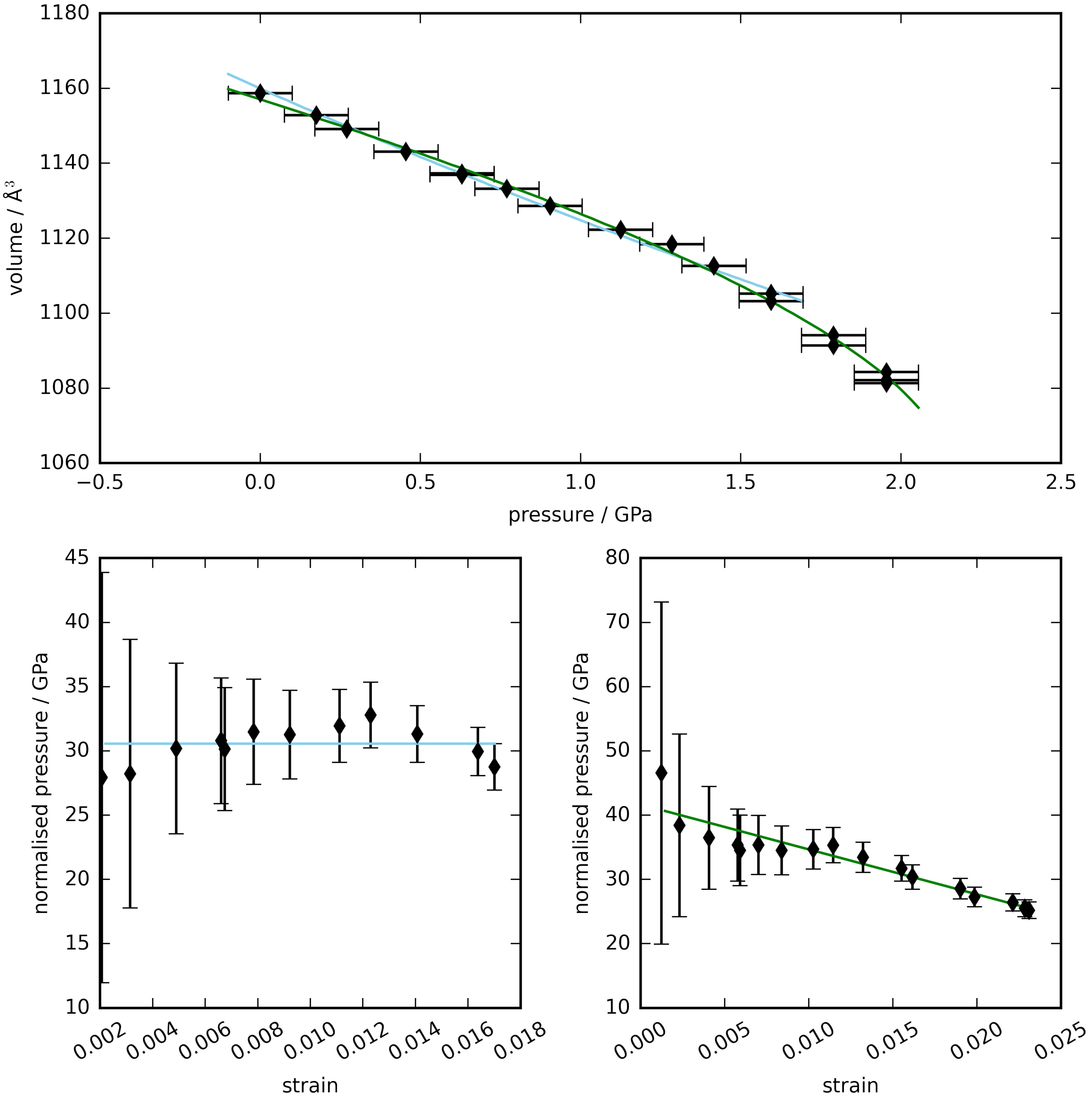

Figure S18: (top) Fits to experimental unit cell volumes using second-order (blue) and third-order (green) Birch-Murnaghan equation of state for the ambient phase of CsMnCo. The normalised pressure *vs.* Eulerian strain for (left) the second-order and (right) the third-order equation of state.

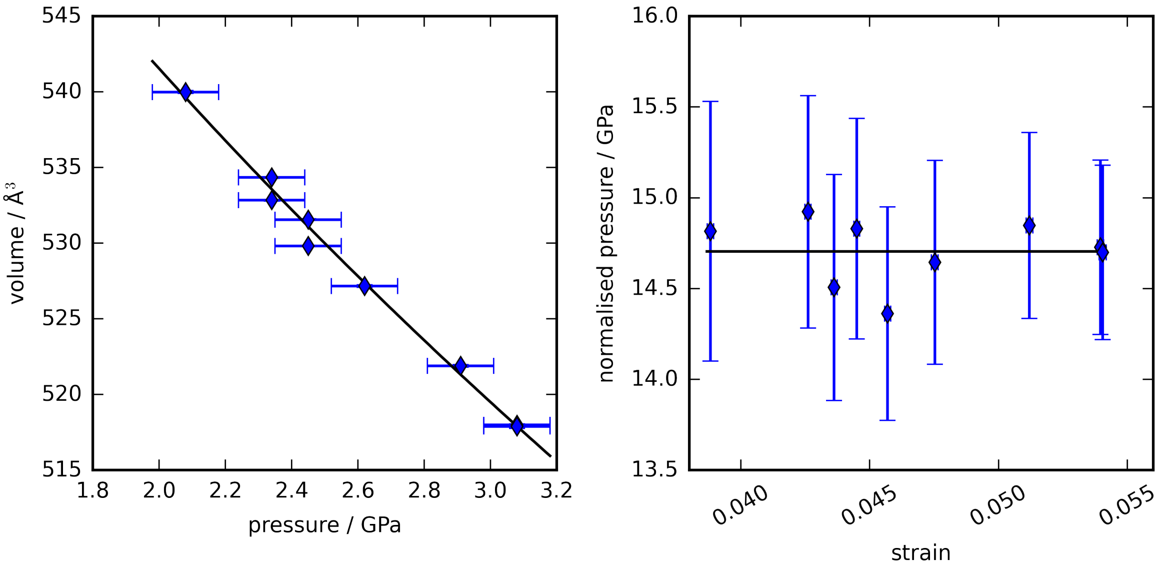

Figure S19: (left) Fits to experimental unit cell volumes using second-order Birch-Murnaghan equation of states and (right) the normalised pressure *vs.* Eulerian strain for the high-pressure phase CsMnCo-II.

6 Crystallographic details of new phases

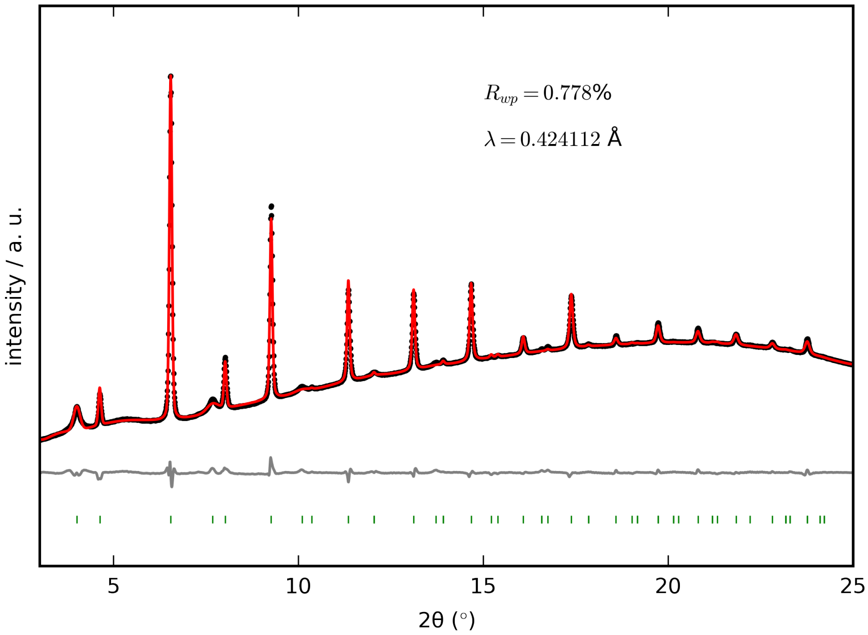

Figure S20: The Rietveld fit of the ambient phase of $CsMnCo(CN)_6$ in $F\bar{4}3m$. Experimental data are shown in black, the fit in red, the residuals in grey and allowed reflections indicated by vertical bars.

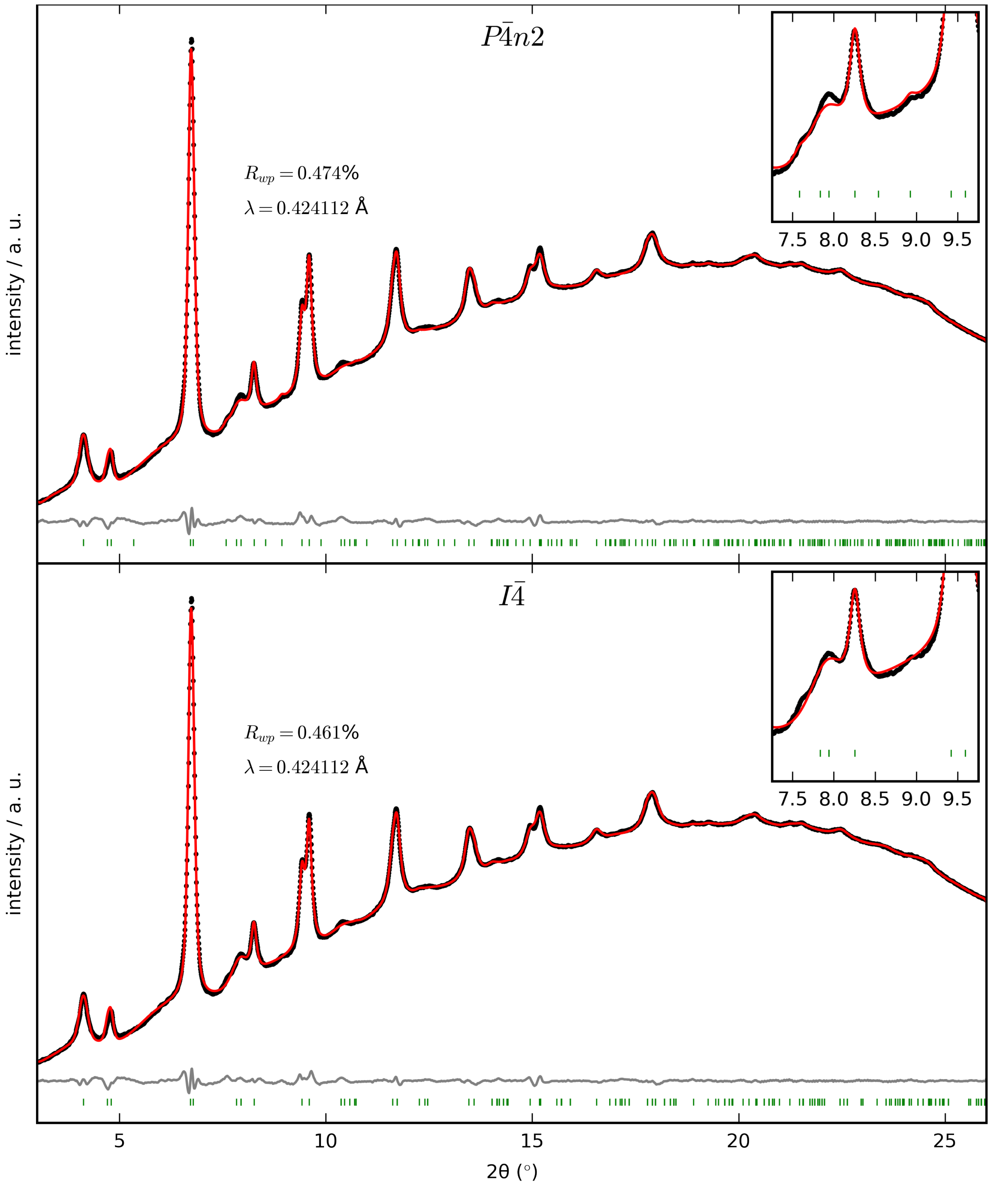

Figure S21: The Rietveld fit of CsMnCo-II in $P4n2$ and $I\bar{4}$ at 2.45 GPa. The experimental data are shown in black, the fit in red, the residuals in grey and allowed reflections indicated by vertical bars.

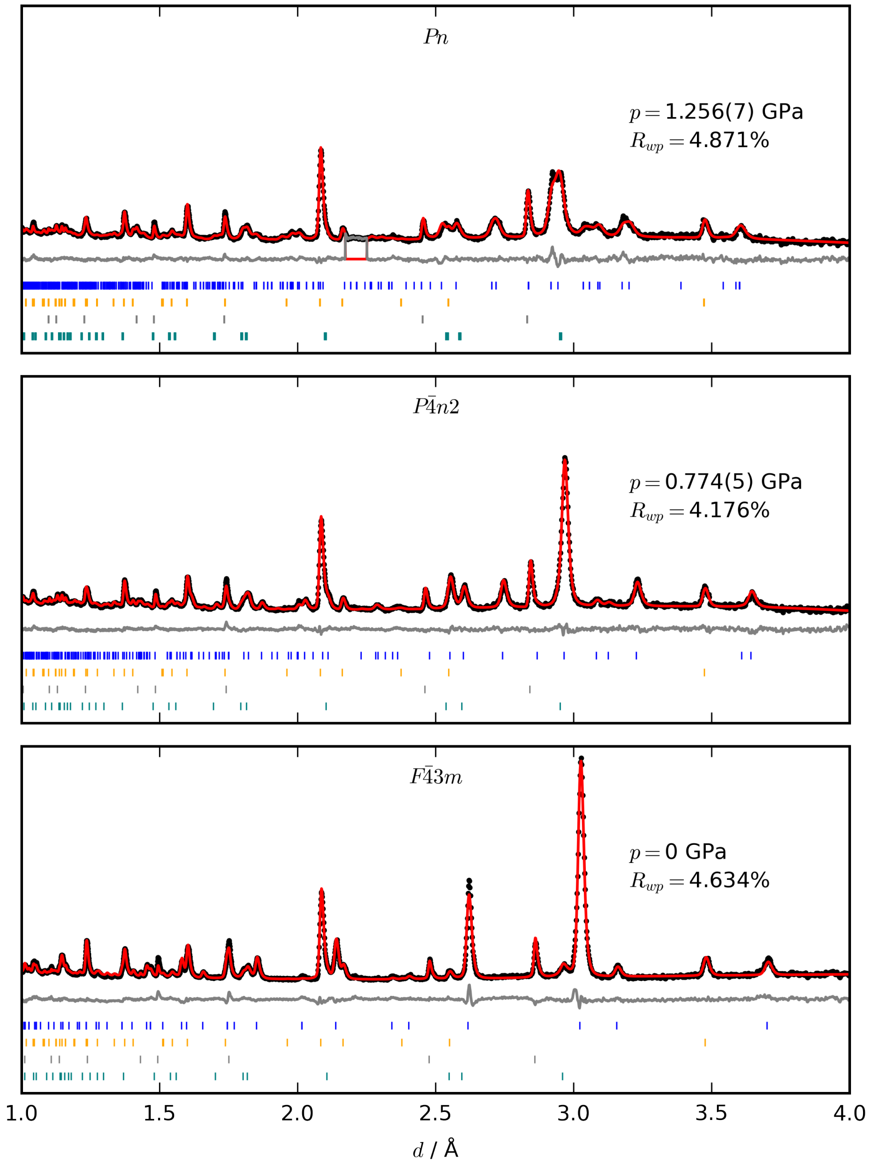

Figure S22: The Rietveld fits of the three phases of RbMnCo. Experimental data are shown in black, the fitted Rietveld profile in red, residuals in grey and allowed reflections indicated by vertical bars. Colour code: RbMnCo (blue), alumina (yellow), lead (grey), and zirconia (teal). The region at 2.2 Å contains an additional reflection from the anvil material and was excluded from the refinement of the monoclinic phase.

Table S10: Crystallographic details determined by Rietveld refinement of PXRD data for CsMnCo at ambient conditions. Data collected at I15, Diamond Light Source. CSD 2048874.

Space group		$F\bar{4}3m$			
a / Å		10.50332(9)			
V / Å^3		1158.72(3)			
Z		4			
R_{wp}		0.778%			
Atom	occ	x	y	z	B_{iso}* / Å^2
Cs	0.729(2)	0.25	0.25	0.25	2.14(4)
Cs	0.271(2)	0.75	0.25	0.25	2.14
Mn	1	0	0	0.5	2.14
Co	1	0	0	0	2.14
C	1	0	0	0.195(7)	2.14
N	1	0	0	0.302(6)	2.14

* B_{iso} constrained to be equal for all atoms.

Table S11: Crystallographic details determined by Rietveld refinement of PXRD data for CsMnCo-II at 2.45 GPa. Data collected at I15, Diamond Light Source. CSD 2048877.

Space group		$P\bar{4}n2$			
a / Å		7.1722(2)			
c / Å		10.3304(6)			
V / Å^3		531.40(4)			
Z		2			
R_{wp}		0.474%			
Atom	occ	x	y	z	B_{iso}* / Å^2
Cs	0.734(2)	0	0.5	0.25	2.03(6)
Cs	0.266(2)	0	0.5	0.75	2.03
Co	1	0	0	0.5	2.03
Mn	1	0	0	0	2.03
N	1	0.739(4)	0.840(4)	0.0279(16)	2.03
N	1	0	0	0.204(6)	2.03
C	1	0.694(6)	0.693(6)	0.003(6)	2.03
C	1	0	0	0.312(7)	2.03

* B_{iso} constrained to be equal for all atoms.

Table S12: Crystallographic details determined by Rietveld refinement of PXRD data for RbMnCo at ambient conditions. Data collected at PEARL, ISIS. CSD 2048876.

Space group		$F\bar{4}3m$			
a / Å		10.47265(11)			
V / Å^3		1148.60(4)			
Z		4			
R_{wp}		4.634%			
Atom	occ	x	y	z	B_{iso}* / Å^2
Rb	0.61993	0.25	0.25	0.25	1.94(6)
Rb	0.25007	0.75	0.75	0.75	1.94
Co	0.91	0.5	0.5	0.5	1.94
Mn	1	0	0	0	1.94
N	0.91	0.2096(3)	0	0	1.94
C	0.91	0.3231(4)	0	0	1.94

* B_{iso} constrained to be equal for all atoms.

Table S13: Crystallographic details determined by Rietveld refinement of PXRD data for RbMnCo-II at 0.774(5) GPa. Data collected at PEARL, ISIS. CSD 2048878.

Space group		$P\bar{4}n2$			
a / Å		7.2191(3)			
c / Å		10.4037(8)			
V / Å^3		542.19(6)			
Z		2			
R_{wp}		4.176%			
Atom	occ	x	y	z	B_{iso}* / Å^2
Rb	0.61993	0	0.5	0.25	1.45(10)
Rb	0.25007	0	0.5	0.75	1.45
Co	0.91	0	0	0.5	1.45
Mn	1	0	0	0	1.45
N	0.91	0.7359(11)	0.8425(11)	0.0175(11)	1.45
N	0.91	0	0	0.2114(11)	1.45
C	0.91	0.6516(14)	0.7125(14)	−0.001(2)	1.45
C	0.91	0	0	0.3210(15)	1.45

* B_{iso} constrained to be equal for all atoms.

Table S14: Crystallographic details determined by Rietveld refinement of PXRD data for RbMnCo-III at 1.256(7) GPa. Data collected at PEARL, ISIS. CSD 2048875.

Space group	Pn		
a / Å	7.0855(8)		
a / Å	7.1789(8)		
c / Å	10.2949(15)		
β / °	90.01(5)		
V / Å^3	523.66(11)		
Z	2		
R_{wp}	4.871%		

Atom	occ	x	y	z
Rb	0.61993	0	0.25	0.75
Rb	0.25007	0	0.25	0.25
Co	0.91	0	0.75	0.5
Mn	1	0	0.75	0
N	0.91	0.7243(11)	−0.0922(11)	−0.0291(6)
N	0.91	0.3422(11)	0.5257(11)	0.4709(6)
N	0.91	0.2757(11)	0.5922(11)	0.0291(6)
N	0.91	0.6578(11)	−0.0257(11)	0.5291(6)
N	0.91	0.0582(13)	0.75	0.7832(8)
N	0.91	−0.0582(13)	0.75	0.2168(8)
C	0.91	0.6451(15)	0.0272(15)	−0.0118(10)
C	0.91	0.2228(15)	0.6049(15)	0.4882(10)
C	0.91	0.3549(15)	0.4728(15)	0.0118(10)
C	0.91	0.7772(15)	0.8951(15)	0.5118(10)
C	0.91	0.024(2)	0.75	0.6840(9)
C	0.91	−0.024(2)	0.75	0.3160(9)

7 Pressure-induced amorphisation (PIA) in MnPt·nD$_2$O and Mn[Co]$_{0.67}$·nD$_2$O

As noted in the main text, MnPt·nD$_2$O and Mn[Co]$_{0.67}$·nD$_2$O undergo reversible pressure-induced amorphisation (PIA) in our variable-pressure neutron diffraction experiment (PEARL, ISIS), but the analogous hydrogenous samples (MnPt·nH$_2$O and Mn[Co]$_{0.67}$·nH$_2$O) remain crystalline when measured using variable-pressure X-ray diffraction (ID15B, ESRF).[S1] PIA was identified by the broadening and eventual disappearance of Bragg reflections in the diffraction pattern; this does not indicate the nature of the high-pressure phase, but rather suggests loss of crystallinity and the formation of an amorphous or highly-disordered material. In the previous XRD experiment—carried out by the present authors—both samples undergo a structural phase transition at 1.31(10) GPa and 1.46(13) GPa respectively. The nature of the high-pressure phases, compressibilities *etc.* of those phase transitions were fully rationalised by symmetry mode analysis and the onset of octahedral tilting.[S1]

PIA can be defined as the formation of an amorphous solid directly from crystalline material through the application of pressure, at a temperature below the melting point or glass transformation range, *i.e.* PIA is a metastable melting event [Fig. S23].[S10, S11] Thus, PIA may occur if a crystalline phase transition is kinetically impeded, such that volume contraction is allowed by the formation of a high-density amorphous phase rather than a high-density crystalline phase, as might otherwise (at higher temperature) be observed. The amorphous state might also show a larger compressibility than the associated crystalline state, providing a thermodynamic driving force for this transition. Note that PIA as a melting event is a reversible process.

PIA has been reported in several framework materials, including NTE and cyanide frameworks, such as the PBA Fe[Co(CN)]$_6$.[S12] The presence of low-energy phonon modes in cyanide materials and other framework materials has been proposed as the mechanism responsible for both NTE and PIA, giving a general correspondence between the two phenomena.[S13, S14] In the study of Fe[Co(CN)]$_6$,[S12] the PIA was investigated using both angle- and energy-dispersive XRD, Raman and infrared spectroscopy, revealing irreversible amorphisation above 10 GPa. Recovery of the material following compression indicated sample decomposition to amorphous carbon nitride and metallic iron and cobalt. In addition, X-ray amorphisation of framework cyanides has been reported, with the NTE material Zn(CN)$_2$ amorphising as a function of X-ray exposure.[S7] So, while PIA is known in framework materials, the reason for reversible amorphisation using neutron diffraction (but not XRD) in this case is not clear.

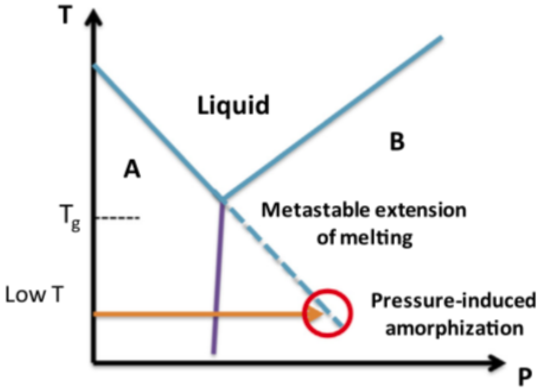

Figure S23: Schematic depiction of PIA as a metastable melting event. A low-pressure phase A has a negative melting slope, intersected by a first-order phase transition into a dense phase B at high pressure. If the compression is carried out at sufficiently low temperature such that the crystalline phase transition is kinetically impeded, the system can intersect the metastable extension of the melting line of A at some temperature below the glass transition (T_g). This results in a direct transformation of the metastably compressed crystal into an amorphous solid, that can be mapped on to a high-P, low-T version of the glassy state derived from the supercooled liquid. Figure reproduced with permission from Ref. S 10.

There are several important differences in our high-pressure neutron and X-ray diffraction experiments that *may* give rise to this contrasting behaviour, in addition to known experimental variations in high-pressure experiments. In particular:

- The pressure was generated using a diamond-anvil cell (DAC) in the XRD experiments, whereas a Paris-Edinburgh (P-E) press was used in the neutron diffraction. Consequently the sample volume is much smaller (1–10 mg) in our XRD (DAC) experiment compared to neutron diffraction experiments in the P-E press (>100 mg).
- One DAC experiment was carried out on a single crystal, while all other experiments were performed on polycrystalline samples.
- For all DAC experiments, Daphne 7373 was used as the pressure-transmitting medium (PTM), whereas the P-E experiments were repeated with both deuterated Fluorinert (84/87) and 1:1 pentane:isobutane mixture.
- All XRD samples were hydrated, whereas D_2O was used for our neutron diffraction experiments due to the large incoherent neutron scattering length of hydrogen.

- Data collection times were longer for our neutron diffraction (2–4 hours per data point) compared to (synchrotron) XRD experiments (<1 minute per data point).
- In all experiments, the pressure is not homogenous within the enclosed volume giving rise to strain gradients that may cause inconsistent results.

The majority of these experimental differences are unavoidable given the experimental constraints of each technique (type of pressure device, collection times, deuteration, sample size), without carrying out dedicated experiments that are beyond the scope of this work. To preclude an experimental anomaly, *e.g.* an unusually high strain field, poor mixing between sample and PTM, the neutron diffraction experiments were repeated with different PTMs, resulting in broadly similar results. Unfortunately a deuterated version of Daphne 7373 (a commercial lubricant composed mostly of olefin oligomer) is not readily available, so the experiments could not be carried out with the same PTM. However, in the pressure range of interest all three PTMs used are hydrostatic and non-penetrating,[S15] so the reproducibility of the amorphisation experiment suggests this is not an anomaly.

We consider three possible factors that might account for these results: (i) increased grain–grain interactions in the larger sample volume and higher sample:PTM ratio in the P-E press setup, (ii) an isotope effect due to the difference between H_2O and D_2O interactions with the framework, and (iii) localised heating effect in XRD experiments that are absent in the neutron diffraction experiments. Of these we believe the first is most likely, as grain size and grain–grain interactions are believed to affect the onset of amorphisation due to an increase in nucleation sites for the amorphisation process.[S10] This was observed in a previous study on the phase transition behaviour of single crystals of $Ca(OH)_2$ that undergo a crystal–crystal phase transition at 6 GPa and remain crystalline up to 20 GPa, compared to PIA in polycrystalline $Ca(OH)_2$ at ~11 GPa.[S16] Other studies do suggest, in contrast, that smaller crystallite sizes might lead to higher mechanical stability. As an example, boron carbide exhibits different mechanical properties depending on processing, with ultrafine samples having a lower propensity for amorphisation.[S17] Strain rate effects were also investigated as a contributing factor in this study.[S17] However, although the strain rate is different between our experiments, it is not particularly high. Therefore to account for the present results, grain-grain (interfacial) interactions must be significantly larger in the larger sample volume within the P-E press compared to the DAC.

Solvent–framework interactions are known to affect amorphisation. For example, the inclusion of water in zeolite AlPO4-17[S18] and solvent in the MOF ZIF-8[S19] stabilise these materials

against amorphisation. Furthermore, penetrating PTMs can be included within pores during a variable-pressure experiment, *e.g.* Al-CAU-13 MOF,[S20] stabilising the material compared to similar compressions in non-penetrating media, therefore preventing PIA. In the present case, as all samples are hydrated and measured with non-penetrating media, so the only factor that could change the stability between samples is the small possible difference in hydrogen/deuterium-bonding and H_2O/D_2O-framework interaction strength.

As PIA arises from a kinetically impeded phase transition, both the temperature and time of the experiment could change the observed (kinetic/thermodynamic) phase behaviour.[S10] For this to explain the difference between our XRD and neutron diffraction experiments, the thermal energy from X-ray exposure—estimated to increase sample temperature by up to 8 K at third generation synchrotron sources[S21]—would have to be sufficient to overcome this kinetic barrier in a relatively short exposure. This temperature effect would be in competition with the more well-known beam damage processes that lead to amorphisation with exposure,[S7] counter to our results. The relatively small and potentially very localised temperature increase may not be sufficient to significantly alter the kinetics of the phase transition. Furthermore, the longer experiment times of our neutron experiments might favour a thermodynamic rather than kinetic compression product. While we have attempted to provide possible explanations for the different behaviour of equivalent samples between our experiments, further work is required to conclusively rationalise this.

8 References

(S1) Boström, H. L. B.; Collings, I. E.; Cairns, A. B.; Romao, C. P.; Goodwin, A. L. High-pressure behaviour of Prussian blue analogues: interplay of hydration, Jahn-Teller distortions and vacancies. *Dalton Trans.* **2019**, *48*, 1647–1655.

(S2) Cliffe, M. J.; Goodwin, A. L. PASCal: A principal axis strain calculator for thermal expansion and compressibility determination. *J. Appl. Cryst.* **2012**, *45*, 1321–1329.

(S3) Birch, F. Finite elastic strain of cubic crystals. *Phys. Rev.* **1947**, *71*, 809–824.

(S4) Murnaghan, F. D. The compressibility of media under extreme pressures. *Proc. Natl. Acad. Sci. U.S.A* **1944**, *30*, 244–247.

(S5) Angel, R. J.; Gonzalez-Platas, J.; Alvaro, M. EosFit7c and a Fortran module (library) for equation of state calculations. *Z. Kristallogr.* **2014**, *229*, 405–419.

(S6) Collings, I. E.; Cairns, A. B.; Thompson, A. L.; Parker, J. E.; Tang, C. C.; Tucker, M. G.;

Catafesta, J.; Levelut, C.; Haines, J.; Dmitriev, V.; Pattison, P.; Goodwin, A. L. Homologous Critical Behavior in the Molecular Frameworks $Zn(CN)_2$ and Cd(imidazolate)$_2$. *J. Am. Chem. Soc.* **2013**, *135*, 7610–7620.

(S7) Lapidus, S. H.; Halder, G. J.; Chupas, P. J.; Chapman, K. W. Exploiting high pressures to generate porosity, polymorphism, and lattice expansion in the nonporous molecular framework $Zn(CN)_2$. *J. Am. Chem. Soc.* **2013**, *135*, 7621–7628.

(S8) Wei, Z.; Tan, L.; Cai, G.; Phillips, A. E.; da Silva, I.; Kibble, M. G.; Dove, M. T. Colossal pressure-induced softening in scandium fluoride. *Phys. Rev. Lett.* **2020**, *124*, 255502.

(S9) Fang, H.; Dove, M. T. Pressure-induced softening as a common feature of framework structures with negative thermal expansion. *Phys. Rev. B* **2013**, *87*, 214109.

(S10) Machon, D.; Meersman, F.; Wilding, M. C.; Wilson, M.; McMillan, P. F. Pressure-induced amorphization and polyamorphism: Inorganic and biochemical systems. *Prog. Mater. Sci.* **2014**, *61*, 216–282.

(S11) Sharma, S. M.; Sikka, S. K. Pressure induced amorphization of materials. *Prog. Mater. Sci.* **1996**, *40*, 1–77.

(S12) Catafesta, J.; Haines, J.; Zorzi, J. E.; Pereira, A. S.; Perottoni, C. A. Pressure-induced amorphization and decomposition of $Fe[Co(CN)_6]$. *Phys. Rev. B* **2008**, *77*, 064104.

(S13) Keen, D. A.; Goodwin, A. L.; Tucker, M. G.; Dove, M. T.; Evans, J. S. O.; Crichton, W. A.; Brunelli, M. Structural description of pressure-induced amorphization in ZrW_2O_8. *Phys. Rev. Lett.* **2007**, *98*, 225501.

(S14) Speedy, R. J. Models for the amorphization of compressed crystals. *J. Phys. Condens. Matter* **1996**, *8*, 10907.

(S15) Klotz, S.; Chervin, J.-C.; Munsch, P.; Le Marchand, G. Hydrostatic limits of 11 pressure transmitting media. *J. Phys. D: Appl. Phys.* **2009**, *42*, 075413.

(S16) Ekbundit, S.; Leinenweber, K.; Yarger, J. L.; Robinson, J. S.; Verhelst-Voorhees, M.; Wolf, G. H. New high-pressure phase and pressure-induced amorphization of $Ca(OH)_2$: Grain size effect. *J. Solid State Chem.* **1996**, *126*, 300–307.

(S17) DeVries, M.; Pittari III, J.; Subhash, G.; Mills, K.; Haines, C.; Zheng, J. Q. Rate-Dependent Mechanical Behavior and Amorphization of Ultrafine-Grained Boron Carbide. *J. Am. Ceram. Soc.* **2016**, *99*, 3398–3405.

(S18) Alabarse, F. G.; Joseph, B.; Lausi, A.; Haines, J. Effect of H_2O on the Pressure-Induced Amorphization of Hydrated $AlPO_4$-17. *Molecules* **2019**, *24*, 2864.

(S19) Poryvaev, A. S.; Polyukhov, D. M.; Fedin, M. V. Mitigation of Pressure-Induced Amor-

phization in Metal–Organic Framework ZIF-8 upon EPR Control. *ACS Appl. Mater. Interfaces* **2020**, *12*, 16655–16661.

(S20) Wharmby, M. T.; Niekiel, F.; Benecke, J.; Waitschat, S.; Reinsch, H.; Daisenberger, D.; Stock, N.; Yot, P. G. Influence of Thermal and Mechanical Stimuli on the Behavior of Al-CAU-13 Metal–Organic Framework. *Nanomaterials* **2020**, *10*, 1698.

(S21) Wallander, H.; Wallentin, J. Simulated sample heating from a nanofocused X-ray beam. *J. Synchrotron Radiat.* **2017**, *24*, 925–933.
